# Disease severity and mortality in Alzheimer's disease: an analysis using the U.S. National Alzheimer’s Coordinating Center Uniform Data Set

**DOI:** 10.1186/s12883-023-03353-w

**Published:** 2023-08-14

**Authors:** Valerie Crowell, Adriana Reyes, Summera Qiheng Zhou, Maria Vassilaki, Sandro Gsteiger, Anders Gustavsson

**Affiliations:** 1 F. Hoffmann-La Roche Ltd, Basel, Switzerland; 2 Genesis Research, Hoboken, NJ USA; 3 Department of Quantitative Health Sciences, Mayo Clinic, Rochester, MN USA; 4 Quantify Research, Stockholm, Sweden; 5 Department of Neurobiology, Care Sciences and Society, Karolinska Institutet, Stockholm, Sweden

**Keywords:** Mortality, Alzheimer, Mild cognitive impairment, Prodromal AD, Predementia, Comorbidity, Institution

## Abstract

**Background:**

Evidence on the relative risk of death across all stages of Alzheimer’s disease (AD) is lacking but greatly needed for the evaluation of new interventions. We used data from the Uniform Data Set (UDS) of the National Alzheimer’s Coordinating Center (NACC) to assess the expected survival of a person progressing to a particular stage of AD and the relative risk of death for a person in a particular stage of AD compared with cognitively normal (CN) people.

**Methods:**

This was a retrospective observational cohort study of mortality and its determinants in participants with incident mild cognitive impairment (MCI) due to AD or AD dementia compared with CN participants. Overall survival and hazard ratios of all-cause mortality in participants ≥ 50 years of age with clinically assessed or diagnosed MCI due to AD, or mild, moderate, or severe AD dementia, confirmed by Clinical Dementia Rating scores, versus CN participants were estimated, using NACC UDS data. Participants were followed until death, censoring, or until information to determine disease stage was missing.

**Results:**

Aged between 50 and 104 years, 12,414 participants met the eligibility criteria for the study. Participants progressing to MCI due to AD or AD dementia survived a median of 3–12 years, with higher mortality observed in more severe stages. Risk of death increased with the severity of AD dementia, with the increase significantly higher at younger ages. Participants with MCI due to AD and CN participants had a similar risk of death after controlling for confounding factors.

**Conclusions:**

Relative all-cause mortality risk increases with AD severity, more so at younger ages. Mortality does not seem to be higher for those remaining in MCI due to AD. Findings might imply potential benefit of lower mortality if preventing or delaying the progression of AD is successful, and importantly, this potential benefit might be greater in relatively younger people. Future research should replicate our study in other samples more representative of the general US population as well as other populations around the world.

**Supplementary Information:**

The online version contains supplementary material available at 10.1186/s12883-023-03353-w.

## Background

The burden of Alzheimer’s disease (AD) is enormous, with the latest global estimates suggesting that up to 100 million people could be affected when including those in the early symptomatic stages of the disease (i.e., mild cognitive impairment [MCI] due to AD and AD dementia) [1]. AD is associated with impaired quality of life and premature death [2, 3], substantial burden to family care partners [4], and large costs to society [5]. As global numbers are expected to rise, policymakers and drug developers are determined to find a way to prevent or slow disease progression in AD. Improving our understanding of the relationship between AD and mortality is crucial to achieving this in two key ways. Firstly, mortality is a major concern for people living with AD and their families [6, 7]. Secondly, the relationship between AD and mortality is expected to have a major impact on the long-term consequences and value of efforts to prevent or delay the disease, since treatment benefit would be determined by a therapy’s ability to both reduce morbidity and extend life [8]. At present we have limited knowledge of precisely how mortality is affected by AD, and specifically by AD progression and disease severity. This limits our ability to assess the value of interventions and thus make informed policy decisions.

Dementia is the fifth leading cause of death worldwide, accounting for 2.4 million deaths worldwide in 2016 [3]. The median survival after a dementia diagnosis has been estimated to be between 3 and 7 years [9], suggesting a loss of approximately 9–10 years of life for a 70-year-old person with AD dementia [10]. The available literature consistently demonstrates reductions in life expectancy in AD and dementia, but there are several confounding factors that complicate the picture [9, 11, 12]. In addition to age, key risk factors for death in AD populations include male sex [13–17], advanced disease severity [13–17] and comorbidities (in particular diabetes [13, 14, 18] but also hypertension [18]), coronary artery disease (CAD) [14], and cerebrovascular disease [15, 19]. Additional factors found to be associated with an increased risk of death in some AD studies include being White [15, 20, 21], lower levels of education [17, 19], being underweight (according to body mass index [BMI]) [22], and apolipoprotein E (*APOE*) ε4-positivity (albeit only in men) [23]. Living in an institutional care setting has also been shown to be associated with an increased risk of death in people with dementia of any cause [24].

Evidence on how disease severity impacts the risk of death is essential for disease models used to predict long-term outcomes and evaluate new interventions. Such long-term effects are typically not possible to assess in randomized controlled trials because the length of follow-up is too short; therefore, longer-term observational studies are also needed [8]. There have been a number of such studies in people with AD [13, 14, 25] and all-cause dementia populations [24–26] but these can have significant limitations, especially when evaluating interventions in early disease. Firstly, studies on predementia stages of disease are lacking and rarely make comparisons with cognitively normal (CN) participants [13, 14, 24, 25]. Secondly, some studies perform their analyses on prevalent cases [24, 25], thereby lacking a clinically relevant starting point for the exposure and potentially introducing survival bias [12]. Thirdly, some studies define disease stage based on single outcome measures (e.g., cognition) [24], thereby omitting other important symptoms such as functional impairment and behavior. Finally, some studies run their analyses on cohorts combining several dementia etiologies [24, 26] or do not account for important confounding factors (e.g., comorbidities) [25, 26], both of which limit the generalizability of their findings to specific populations.

This study was therefore performed on a well-characterized US population, enabling up to 15 years of follow-up of participants across all clinical stages of AD. We had two main research questions: 1) What is the expected survival of a person progressing to a particular stage of AD? and 2) What is the relative risk of death of a person in a particular stage of AD compared with CN people?

## Methods

### Study design

This was a retrospective observational cohort study of mortality, and the factors that affect the risk of death, in participants with incident MCI due to AD or AD dementia compared with CN participants. Individual-level data were sourced from the Uniform Data Set (UDS) of the National Alzheimer’s Coordinating Center (NACC) [27]. Study participants were identified in the NACC UDS at any time during the study period September 1, 2005 to March 1, 2021.

### Data sources

The NACC UDS is a large, longitudinal, and well-structured data set of participants with varying cognitive statuses, ranging from normal cognition to MCI and demented. An additional category, “impaired not MCI”, exists for subjects who are cognitively impaired but do not meet the criteria for MCI [27]. Standardized data have been collected prospectively at Alzheimer’s Disease Research Centers (ADRCs) throughout the US since 2005, funded by the National Institute on Aging (NIA). The data used in this study included all visits for the subset of UDS participants who consented to allow the sharing of research data with commercial entities. This analysis used data from 33 ADRCs.

After the initial UDS visit, individuals had an annual follow-up visit until death or dropout. The data collected included socio-demographics, family and health history, neurologic exam findings, functional status, clinical diagnosis, neuropsychologic test results, imaging availability, and *APOE* status.

Disease severity measures of relevance used in this study included the Clinical Dementia Rating (CDR^®^) Dementia Staging Instrument [28] (providing both Global Scores [CDR-GS] and Sum of Boxes [CDR-SB]), the Mini-Mental State Examination (MMSE) [29] and the Montreal Cognitive Assessment (MoCA) [30] for cognitive impairment, the Functional Activities Questionnaire (FAQ) [31] for functional impairment, and the Neuropsychiatric Inventory Questionnaire (NPI-Q) [32] for behavioral and psychiatric symptoms.

Deaths were reported by the ADRCs via a specific milestones form, and therefore these were only for participants who were actively followed.

### Eligibility criteria and cohorts

Eligible participants had an index visit at 50 years old or older and contributed an observation time of at least one day. At their index visit, participants were classified into one of three cohorts: CN, MCI due to AD, and AD dementia; see Additional file 1 for details on variables and cutoff scores for each cohort.

CN participants had a clinical diagnosis of normal cognition confirmed by CDR scores at index, and no prior record of impairment at any pre-index visit. Since clinical diagnosis of normal cognition is based on the absence of symptoms, subjects in this cohort could be in the preclinical stages of AD. Participants with MCI due to AD had a clinical diagnosis of amnestic or non-amnestic MCI confirmed by CDR scores at index, and no prior record of more severe impairment. To limit to incident cases of AD, participants were further required to have a pre-index visit with normal cognition or impaired not MCI, confirmed by CDR scores within 15 months before the index date. They were also required to have a record of AD etiology at index or after, and no record of non-AD etiologic diagnosis potentially causing cognitive impairment before or at index, with the exception of anxiety and depression, because these have been suggested to be possible first signs of AD [33, 34] (see Additional file 1).

Participants with AD dementia had a clinical diagnosis of dementia, confirmed by CDR and MMSE scores at index, and no prior record of such impairment. They were required to have a pre-index visit with normal cognition, impaired not MCI, or MCI, confirmed by CDR scores within 15 months before the index date. They were also required to have a presumptive primary etiologic diagnosis of AD at index, and no record of a non-AD etiologic diagnosis potentially causing cognitive impairment before or at index, with the exception of anxiety and depression. Finally, to enable further dementia staging, participants with AD dementia were required to have complete MMSE or MoCA scores, complete NPI-Q scores, and at least five completed tasks for the FAQ scale at index (see Additional file 1).

Due to limitations in data availability, biomarker confirmation of AD etiology was not possible, except for in a subset of participants with biomarker-positive MCI due to AD (*n* = 47) who were considered in a sensitivity analysis. Biomarker-positivity was defined as abnormally elevated amyloid on positron emission tomography or abnormally low amyloid in cerebrospinal fluid.

Participants with AD dementia were further classified into one of three disease severity stages (mild, moderate, and severe), according to a previously published model using scores on cognitive, behavioral, and functional symptom domains as measured by the MMSE, NPI-Q, and FAQ scales [35] (see Additional file 1).

Individual participants could contribute observation time to several cohorts upon disease progression if they met the corresponding eligibility criteria. Back-transitions to less severe stages of AD were disregarded to simplify the analysis, and as they are likely temporary in most cases (Supplementary Table 2) [36, 37].

### Follow-up

Participants were followed until death, censoring (either at the end of the study period or due to discontinuation or loss to follow-up), or until information to determine disease stage was missing; participants who progressed to a more severe disease stage were included in the subsequent cohort if they met the eligibility criteria (see Additional file 1 and Supplementary Table 1).

### Outcomes

Key outcomes included overall survival (median time in years from progression to a particular stage, or study entry for CN participants, to date of death), and hazard ratios (HRs) for different disease stages in comparison with CN participants, with or without adjustment for age, and other potential confounding variables. Date of death was recorded with a precision of year and month in the NACC UDS. For our purpose, the date was assumed to be the last day of each month, thereby avoiding records of visits after the assumed date of death, while acknowledging this could potentially lead to overestimating the overall survival by an average of about half a month.

### Predictors and covariates

Age at the start of a particular stage (or study entry for CN) and disease stage (i.e., MCI due to AD, and mild, moderate, or severe AD dementia) were the primary predictors of the analysis, with CN participants as a reference. The selection of additional covariates was guided by a systematic literature review on the determinants of mortality, and they were considered for inclusion if: a) there was convincing evidence of their association with mortality and b) they could be assumed to vary across the different populations that we would like our results to be generalizable to. The following variables were found to meet these criteria: sex [13–17], years of education [17, 19], race/ethnicity [15, 20, 21], BMI [22], *APOE* status [23], presence or history of CAD [14], presence or history of cerebrovascular disease [15, 19], and type of residence [24] (see Additional file 1). In addition, variables for current smoking status and alcohol abuse were considered as they are known to cause premature death [38, 39]. All covariates were recorded at study entry except for age, disease stage, and type of residence, which varied over time and were updated at progression.

### Statistical analysis

To investigate the expected survival of a person progressing to a particular stage of AD, we assessed overall survival separately for each cohort, with Kaplan–Meier estimators reporting median survival time and 95% confidence intervals (CIs) constructed with the Brookmeyer–Crowley method. In this analysis, participants were not censored upon progression but could contribute observation time (and deaths) to multiple cohorts. Therefore, this analysis did not allow for comparison across cohorts but instead shows the expected survival of a person starting in a particular stage of AD irrespective of whether or not they progress. Kaplan–Meier estimators were not stratified by age or other predictors; they describe the overall survival of participants with the age distribution of our sample cohorts.

To assess the relative risk of death of a person in a particular stage of AD compared with CN participants, we fitted Cox proportional-hazards models, adjusting for age and the other covariates. Participants were censored from the stage of AD upon progression, while adding observation time to the new cohort where possible. Five models were considered, including a crude model with disease stage as the only predictor (model 1), a simple model adding age, sex, and a dichotomous variable for up to (or more than) 12 years of education (model 2), an exploratory model including all preselected predictors and covariates (model 3), and an optimized model with covariates selected by the least absolute shrinkage and selection operator (Lasso, model 4), optimized using fivefold nested cross-validation (see Additional file 1) plus sex (based on clinical judgment and interpretability). The fifth model was based on model 4, adding the interaction between disease stage and age because they had large main effects (before fitting the interaction terms) and are of high importance in evaluating the value of a treatment. Other interactions with disease severity were considered including sex, type of residence, years of education, and race/ethnicity, but were not selected either because they were not significant or because the variation in these variables was considered too low. The proportional-hazards model assumption was tested by visual inspection of Schoenfeld residuals. All models were fitted on participants with complete information on the included variables in each particular model, without any imputation.

### Sensitivity analyses

We refitted exploratory model 3 excluding comorbidity variables because it is not clear whether they are on the causal pathway from AD to death (i.e., a mediator) or if they are confounding variables, which, if so, should be controlled [40, 41]. Also, to test whether the COVID-19 pandemic influenced our results, we refitted exploratory model 3 on a data set truncated on March 1, 2020, which is when the COVID-19 lockdown began in the US. We also compared the characteristics of participants with MCI due to AD who had a positive biomarker for amyloid-beta (AMYLPET=1 or AMYLCSF=1) with those in the overall MCI due to AD cohort, but the number of participants was too low for a separate analysis of mortality. The characteristics of participants lost to follow-up were compared with those remaining until death, progression, or data cut.

Finally, in order to test the sensitivity to different classifications of disease severity, we reassigned all participants using an alternative model based on the CDR-SB [42–44], which set cutoffs for CDR-SB at 0.5–4 for MCI due to AD, 4.5–9 for mild AD dementia, 9.5–15.5 for moderate AD dementia, and 16–18 for severe AD dementia [44]. These updated the CDR-GS eligibility criteria and the multi-domain model for AD dementia stages, whereas other criteria were unchanged (Supplementary Table 3). All Cox proportional-hazards models 1 through 5 were refitted using this alternative classification.

## Results

### Participant characteristics

The NACC UDS included 33,874 participants with a visit at ≥ 50 years of age, of whom a total of 12,414 participants met the eligibility criteria for at least one of our cohorts (Table 1, Supplementary Table 3). In 1,045 instances, a participant progressed to a more severe stage and could be included in another cohort, and 917 participants died (Table 1). The mean duration of follow-up ranged from about 2–3 years in AD dementia, up to 3.7 years in MCI due to AD, and 4.4 years in CN, while the maximum follow-up was 15 years (Supplementary Table 1). Most participants with severe AD dementia died during follow-up. In contrast, most participants in the other cohorts either progressed or discontinued before the event of death or end of study.

**Table 1:** Number of participants per cohort, their progressions, deaths, and median Survival (95% CI)

	**Time of entry into cohort**		**Progression from study entry**^**c**^						**All observations from start of stage**^**d**^		
**Cohort**	**Study entry *n***^a^	**Progression *n***^b^	**Did not progress**	**MCI due to AD**	**Mild AD dementia**	**Moderate AD dementia**	**Severe AD dementia**	**Death**	**Total *N***	**Deaths**	**Median survival (95% CI)**
Cognitively normal	11,458	0	10,210	613	29	21	0	585	11,458	686	NA
MCI due to AD	324	613	761	-	53	21	0	102	937	132	12.27 (11.83–NA)
Mild AD dementia	440	82	227	-	-	228	16	51	522	167	6.32 (5.99–7.46)
Moderate AD dementia	187	270	275	-	-	-	64	118	457	162	5.21 (4.88–6.1)
Severe AD dementia	5	80	24	-	-	-	-	61	85	61	3.06 (2.68–3.84)

Data were NA because median survival was not reached during observation period

*AD* Alzheimer's disease, *CI* Confidence interval, *MCI* Mild cognitive impairment, *NA* Not available

^a^Unique participants assigned at the start of the study

^b^Participants progressing from their original cohort, adding observation time to one or more additional cohorts upon progression

^c^Only the immediate progression of a participant is shown on each row

^d^Some participants included in several cohorts if progressing

Participants were aged between 50 and 104 years at study entry, with higher mean ages in the AD cohorts compared with CN (Table 2). About two-thirds (65%) were female, and most were non-Hispanic Whites (75%).

**Table 2:** Participant characteristics by cohort (at start of stage; some participants included in several cohorts if progressing)

	CN	MCI due to AD	Mild AD dementia	Moderate AD dementia	Severe AD dementia	Total (unique participants, at study entry)	Total (overlapping cohorts, at start of stage)
	***n* = 11,458**	***n* = 937**	***n* = 522**	***n* = 457**	***n* = 85**	***n* = 12,414**	***n* = 13,459**
Age at first visit (including pre-index visits)							
Mean (SD)	70.4 (9.15)	75.6 (7.78)	75.5 (7.97)	75.2 (7.64)	73.9 (8.50)	70.8 (9.14)	71.2 (9.14)
Median (Q1, Q3)	70.0 (65.0, 77.0)	76.0 (71.0, 81.0)	76.0 (71.0, 81.0)	75.0 (71.0, 80.0)	75.0 (69.0, 80.0)	71.0 (65.0, 77.0)	71.0 (65.0, 78.0)
Min, Max	38.0, 104	41.0, 102	46.0, 96.0	46.0, 97.0	46.0, 91.0	38.0, 104	38.0, 104
Age at study entry (index visit)							
Mean (SD)	70.5 (9.07)	79.2 (8.05)	78.0 (8.22)	79.0 (8.06)	79.1 (9.07)	71.0 (9.17)	71.7 (9.42)
Median (Q1, Q3)	70.0 (65.0, 77.0)	80.0 (74.0, 85.0)	79.0 (73.0, 84.0)	79.0 (74.0, 85.0)	80.0 (74.0, 85.0)	71.0 (65.0, 77.0)	72.0 (65.0, 78.0)
Min, Max	50.0, 104	51.0, 103	50.0, 97.0	50.0, 98.0	53.0, 96.0	50.0, 104	50.0, 104
Sex, *n* (%)							
Male	3877 (33.8)	390 (41.6)	245 (46.9)	223 (48.8)	40 (47.1)	4337 (34.9)	4775 (35.5)
Female	7581 (66.2)	547 (58.4)	277 (53.1)	234 (51.2)	45 (52.9)	8077 (65.1)	8684 (64.5)
BMI, *n* (%)							
Normal or healthy weight	3685 (32.2)	358 (38.2)	216 (41.4)	195 (42.7)	34 (40.0)	4045 (32.6)	4488 (33.3)
Underweight	122 (1.1)	20 (2.1)	13 (2.5)	9 (2.0)	4 (4.7)	144 (1.2)	168 (1.2)
Overweight	4073 (35.5)	329 (35.1)	197 (37.7)	160 (35.0)	28 (32.9)	4442 (35.8)	4787 (35.6)
Obese	2941 (25.7)	148 (15.8)	66 (12.6)	58 (12.7)	8 (9.4)	3077 (24.8)	3221 (23.9)
Missing	637 (5.6)	82 (8.8)	30 (5.7)	35 (7.7)	11 (12.9)	706 (5.7)	795 (5.9)
Race, *n* (%)							
White	9286 (81.0)	790 (84.3)	453 (86.8)	370 (81.0)	69 (81.2)	10,089 (81.3)	10,968 (81.5)
Black or African American	1668 (14.6)	114 (12.2)	51 (9.8)	60 (13.1)	11 (12.9)	1775 (14.3)	1904 (14.1)
American Indian or Alaska Native	77 (0.7)	2 (0.2)	0 (0)	1 (0.2)	0 (0)	80 (0.6)	80 (0.6)
Native Hawaiian or Other Pacific Islander	9 (0.1)	0 (0)	0 (0)	1 (0.2)	1 (1.2)	10 (0.1)	11 (0.1)
Asian	272 (2.4)	21 (2.2)	12 (2.3)	16 (3.5)	3 (3.5)	298 (2.4)	324 (2.4)
Other	84 (0.7)	5 (0.5)	5 (1.0)	5 (1.1)	1 (1.2)	95 (0.8)	100 (0.7)
Unknown	62 (0.5)	5 (0.5)	1 (0.2)	4 (0.9)	0 (0)	67 (0.5)	72 (0.5)
Hispanic/Latino ethnicity, *n* (%)							
No	10,636 (92.8)	871 (93.0)	497 (95.2)	423 (92.6)	79 (92.9)	11,520 (92.8)	12,506 (92.9)
Yes	767 (6.7)	64 (6.8)	24 (4.6)	31 (6.8)	5 (5.9)	836 (6.7)	891 (6.6)
Unknown	55 (0.5)	2 (0.2)	1 (0.2)	3 (0.7)	1 (1.2)	58 (0.5)	62 (0.5)
Years of education							
Mean (SD)	15.9 (2.94)	15.7 (3.33)	15.5 (3.00)	15.0 (3.35)	15.5 (2.92)	15.9 (2.98)	15.8 (2.99)
Median (Q1, Q3)	16.0 (14.0, 18.0)	16.0 (14.0, 18.0)	16.0 (13.0, 18.0)	16.0 (12.0, 18.0)	16.0 (13.0, 18.0)	16.0 (14.0, 18.0)	16.0 (14.0, 18.0)
Min, Max	0, 29.0	0, 28.0	6.00, 30.0	1.00, 24.0	8.00, 20.0	0, 30.0	0, 30.0
Missing	63 (0.5)	5 (0.5)	1 (0.2)	1 (0.2)	0 (0)	63 (0.5)	70 (0.5)
More than 12 years of education, *n* (%)							
12 years or less	1815 (15.8)	179 (19.1)	120 (23.0)	128 (28.0)	17 (20.0)	2048 (16.5)	2259 (16.8)
More than 12 years	9580 (83.6)	753 (80.4)	401 (76.8)	328 (71.8)	68 (80.0)	10,303 (83.0)	11,130 (82.7)
Missing	63 (0.5)	5 (0.5)	1 (0.2)	1 (0.2)	0 (0)	63 (0.5)	70 (0.5)
Type of residence, *n* (%)							
Single- or multi-family private residence (apartment, condo, house)	10,697 (93.4)	813 (86.8)	466 (89.3)	400 (87.5)	67 (78.8)	11,549 (93.0)	12,443 (92.5)
Retirement community or independent group living	538 (4.7)	85 (9.1)	41 (7.9)	35 (7.7)	11 (12.9)	603 (4.9)	710 (5.3)
Institutionalized	37 (0.3)	12 (1.3)	9 (1.7)	22 (4.8)	7 (8.2)	53 (0.4)	87 (0.6)
Other or unknown	186 (1.6)	27 (2.9)	6 (1.1)	0 (0)	0 (0)	209 (1.7)	219 (1.6)
Currently smoking, *n* (%)							
No	10,917 (95.3)	580 (61.9)	413 (79.1)	318 (69.6)	65 (76.5)	11,640 (93.8)	12,293 (91.3)
Yes	445 (3.9)	15 (1.6)	6 (1.1)	4 (0.9)	0 (0)	460 (3.7)	470 (3.5)
Missing	96 (0.8)	342 (36.5)	103 (19.7)	135 (29.5)	20 (23.5)	314 (2.5)	696 (5.2)
Alcohol abuse, *n* (%)							
Absent	11,002 (96.0)	572 (61.0)	410 (78.5)	309 (67.6)	61 (71.8)	11,710 (94.3)	12,354 (91.8)
Recent/active	47 (0.4)	3 (0.3)	1 (0.2)	4 (0.9)	1 (1.2)	51 (0.4)	56 (0.4)
Remote/inactive	329 (2.9)	16 (1.7)	8 (1.5)	9 (2.0)	3 (3.5)	354 (2.9)	365 (2.7)
Missing	80 (0.7)	346 (36.9)	103 (19.7)	135 (29.5)	20 (23.5)	299 (2.4)	684 (5.1)
*APOE* status, *n* (%)							
No ε4 allele	6636 (57.9)	530 (56.6)	183 (35.1)	156 (34.1)	32 (37.6)	7009 (56.5)	7537 (56.0)
One or two copies of ε4 allele	2945 (25.7)	323 (34.5)	303 (58.0)	273 (59.7)	48 (56.5)	3441 (27.7)	3892 (28.9)
Missing	1877 (16.4)	84 (9.0)	36 (6.9)	28 (6.1)	5 (5.9)	1964 (15.8)	2030 (15.1)
Presence or history of CAD, *n* (%)							
No	10,909 (95.2)	856 (91.4)	487 (93.3)	424 (92.8)	78 (91.8)	11,790 (95.0)	12,754 (94.8)
Yes	527 (4.6)	81 (8.6)	35 (6.7)	33 (7.2)	7 (8.2)	602 (4.8)	683 (5.1)
Missing	22 (0.2)	0 (0)	0 (0)	0 (0)	0 (0)	22 (0.2)	22 (0.2)
Presence or history of CVD, *n* (%)							
No	10,910 (95.2)	871 (93.0)	481 (92.1)	422 (92.3)	78 (91.8)	11,801 (95.1)	12,762 (94.8)
Yes	546 (4.8)	66 (7.0)	41 (7.9)	35 (7.7)	7 (8.2)	611 (4.9)	695 (5.2)
Missing	2 (0.0)	0 (0)	0 (0)	0 (0)	0 (0)	2 (0.0)	2 (0.0)
CDR-GS at index date							
Mean (SD)	0 (0)	0.415 (0.188)	0.662 (0.246)	0.914 (0.402)	1.78 (0.629)	0.0473 (0.181)	0.0968 (0.284)
Median (Q1, Q3)	0 (0, 0)	0.500 (0.500, 0.500)	0.500 (0.500, 1.00)	1.00 (0.500, 1.00)	2.00 (1.00, 2.00)	0 (0, 0)	0 (0, 0)
Min, Max	0, 0	0, 0.500	0.500, 2.00	0.500, 3.00	0.500, 3.00	0, 2.00	0, 3.00
MMSE/MoCA at index date							
Mean (SD)	29.1 (1.38)	27.7 (2.16)	23.9 (1.66)	19.8 (3.15)	12.8 (5.03)	28.7 (2.09)	28.4 (2.81)
Median (Q1, Q3)	30.0 (29.0, 30.0)	28.0 (27.0, 29.0)	24.0 (23.0, 25.0)	20.0 (18.0, 22.0)	14.0 (9.00, 17.0)	29.0 (28.0, 30.0)	29.0 (28.0, 30.0)
Min, Max	16.0, 30.0	18.0, 30.0	21.0, 26.0	10.0, 26.0	2.00, 20.0	4.00, 30.0	2.00, 30.0
Missing	222 (1.9)	56 (6.0)	0 (0)	0 (0)	0 (0)	239 (1.9)	278 (2.1)
FAQ score at index date							
Mean (SD)	0.205 (1.20)	1.95 (3.22)	9.63 (6.14)	16.5 (7.91)	26.7 (4.09)	0.833 (3.18)	1.44 (4.67)
Median (Q1, Q3)	0 (0, 0)	0 (0, 2.50)	9.00 (4.44, 14.0)	17.0 (10.0, 23.0)	28.0 (25.0, 30.0)	0 (0, 0)	0 (0, 0)
Min, Max	0, 30.0	0, 19.0	0, 23.0	0, 30.0	7.50, 30.0	0, 30.0	0, 30.0
Missing	349 (3.0)	31 (3.3)	0 (0)	1 (0.2)	0 (0)	362 (2.9)	381 (2.8)
NPI-Q at index date							
Mean (SD)	0.632 (1.53)	1.54 (2.63)	2.58 (2.47)	4.82 (4.50)	9.98 (6.55)	0.812 (1.86)	0.972 (2.21)
Median (Q1, Q3)	0 (0, 1.00)	0 (0, 2.00)	2.00 (0, 4.00)	3.00 (1.00, 7.00)	9.00 (5.00, 14.0)	0 (0, 1.00)	0 (0, 1.00)
Min, Max	0, 26.0	0, 20.0	0, 12.0	0, 23.0	0, 28.0	0, 26.0	0, 28.0
AD biomarker positive, *n* (%)							
No	732 (6.4)	31 (3.3)	6 (1.1)	12 (2.6)	1 (1.2)	754 (6.1)	782 (5.8)
Yes	210 (1.8)	47 (5.0)	45 (8.6)	42 (9.2)	6 (7.1)	289 (2.3)	350 (2.6)
Missing	10,516 (91.8)	859 (91.7)	471 (90.2)	403 (88.2)	78 (91.8)	11,371 (91.6)	12,327 (91.6)
Number of visits (in this stage)							
Mean (SD)	3.81 (3.16)	3.24 (2.53)	1.90 (1.28)	2.45 (1.54)	1.96 (1.36)	3.72 (3.11)	3.64 (3.06)
Median (Q1, Q3)	3.00 (1.00, 5.00)	2.00 (1.00, 4.00)	1.00 (1.00, 2.00)	2.00 (1.00, 3.00)	1.00 (1.00, 3.00)	3.00 (1.00, 5.00)	3.00 (1.00, 5.00)
Min, Max	1.00, 16.0	1.00, 14.0	1.00, 10.0	1.00, 11.0	1.00, 7.00	1.00, 16.0	1.00, 16.0
Number of visits (overall)							
Mean (SD)	4.93 (3.55)	7.29 (3.44)	5.65 (2.75)	6.28 (2.81)	6.74 (2.46)	4.98 (3.51)	5.18 (3.55)
Median (Q1, Q3)	4.00 (2.00, 7.00)	7.00 (4.00, 10.0)	5.00 (4.00, 7.00)	6.00 (4.00, 8.00)	7.00 (5.00, 8.00)	4.00 (2.00, 7.00)	4.00 (2.00, 7.00)
Min, Max	1.00, 16.0	2.00, 16.0	2.00, 15.0	2.00, 15.0	2.00, 14.0	1.00, 16.0	1.00, 16.0

*AD* Alzheimer's disease, *APOE* Apolipoprotein E, *BMI* Body mass index, *CAD* Coronary artery disease, *CDR-GS* Clinical Dementia Rating – Global Score; *CN* Cognitively normal, *CVD* Cerebrovascular disease, *FAQ* Functional Activities Questionnaire, *MCI* Mild cognitive impairment, *MMSE*, Mini-Mental State Examination, *MoCA* Montreal Cognitive Assessment, *NPI-Q* Neuropsychiatric Inventory Questionnaire, *Q1* First quartile, *Q3* Third quartile, *SD* Standard deviation

### Observed survival

**Fig. 1:**
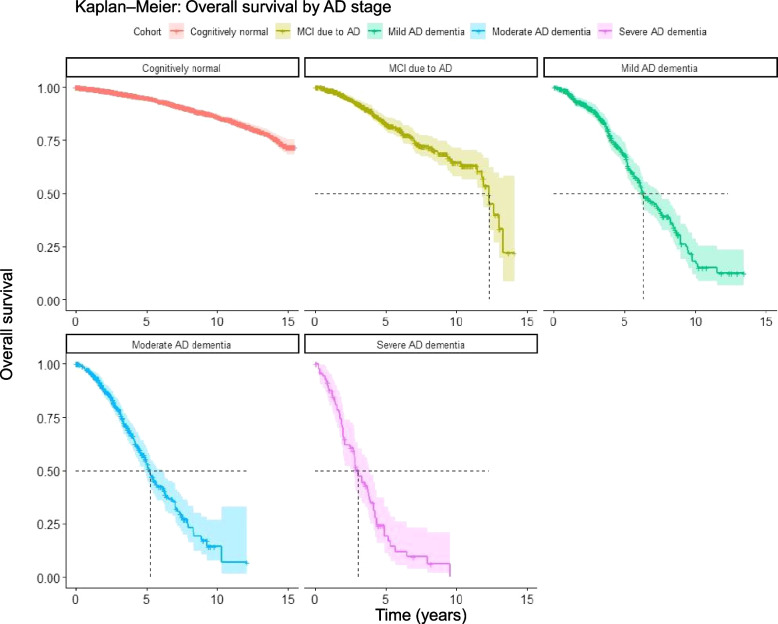
Overall survival of participants progressing to a particular stage of AD. Shaded areas show 95% confidence intervals. Some participants contribute to several stages upon progression. Please note, any comparison across stages underestimates the uncertainty because some participants contribute to several cohorts. The curve of cognitively normal lacks a relevant clinical interpretation as the starting point is their first visit at ≥ 50 years of age. Kaplan–Meier estimators were not stratified by age or other predictors; they describe the overall survival of participants with the age distribution of our sample cohorts. Numbers at risk at 0, 5, 10, and 15 years respectively: CN: 11458, 4185, 1272, 64; MCI due to AD: 937, 249, 50, 0; mild AD dementia: 522, 139, 11, 0; moderate AD dementia: 457, 77, 2, 0; severe AD dementia: 85, 8, 0, 0. *AD Alzheimer’s disease, CN cognitively normal, MCI mild cognitive impairment*

The median overall survival of a person progressing to AD dementia or MCI due to AD was estimated at between 3 and 12 years, with higher mortality in more severe stages (Fig. 1, Table 1). The observation period was too short to estimate median survival in CN participants, as well as for an upper bound of the CI for MCI due to AD.

### Relative risk of death across disease stages

The Cox proportional-hazards models showed a clear pattern of increasing mortality with increasing disease severity (Table 3). The effect sizes were attenuated when controlling for potential confounding factors, but remained statistically significant, with the exception of MCI due to AD compared with CN participants. Additional predictors of higher mortality included: higher age, male sex, residential or institutionalized care setting, presence or history of CAD, currently smoking, and alcohol abuse. Weight categories were defined using BMI [45]; being overweight (BMI = 25.0–29.9) but not obese (BMI ≥ 30.0) predicted lower mortality compared with being underweight (BMI < 18.5) or with BMI in the normal range (BMI = 18.5–24.9); Asian participants had lower relative risk of death compared with White participants, and Hispanic/Latino participants compared with non-Hispanic/Latino participants. Education and *APOE* status were not significant predictors of mortality. Due to missing data, the more comprehensive models were based on fewer observations, while the underlying analysis populations were not substantially different in terms of baseline characteristics (Supplementary Table 8).

**Table 3:** Relative risks of death estimated by Cox proportional-hazards models

	**Model 1**	**Model 2**	**Model 3**	**Model 4**	**Model 5**
	Crude	Parsimonious	Exploratory	Lasso + sex	Age x severity interaction
**Model parameters, hazard ratios (95% CI)**					
**Disease severity**					
CN	ref	ref	ref	ref	ref
MCI due to AD	2.1*** (1.71–2.59)	0.9 (0.72–1.11)	1.04 (0.81–1.34)	0.84 (0.65–1.07)	1.32 (0.08–21.1)
Mild AD dementia	4.92*** (3.68–6.57)	2.05*** (1.52–2.76)	2.36*** (1.66–3.37)	2.08*** (1.49–2.91)	615** (9.70-38-945)
Moderate AD dementia	7.74*** (6.39–9.38)	2.85*** (2.27–3.58)	2.75*** (2.01–3.75)	2.55*** (1.94–3.35)	12827*** (1-102-149-363)
Severe AD dementia	17.2*** (13.5–21.8)	7.04*** (5.32–9.32)	8.04*** (5.51–11.8)	6.19*** (4.36–8.80)	24036*** (1-192-484-606)
**Age at start of stage**		1.11*** (1.10–1.12)	1.11*** (1.10–1.12)	1.1*** (1.09–1.12)	1.13*** (1.11–1.14)
**Sex**					
Male	-	ref	ref	ref	ref
Female	-	0.6*** (0.52–0.68)	0.61*** (0.52–0.72)	0.56*** (0.48–0.65)	0.57*** (0.49–0.66)
**BMI**					
Normal or healthy weight	-	-	ref	ref	ref
Underweight	-	-	1.83* (1.01–3.32)	1.73 (0.93–3.22)	1.48 (0.76–2.89)
Overweight	-	-	0.75** (0.63–0.90)	0.75*** (0.63–0.89)	0.76** (0.64–0.89)
Obese	-	-	1.19 (0.96–1.47)	1.21 (0.99–1.48)	1.24* (1.02–1.51)
**Race**					
White	-	-	ref	-	-
Black or African American	-	-	0.96 (0.73–1.25)	-	-
Asian	-	-	0.23* (0.06–0.87)	-	-
Other	-	-	1.77 (0.59–5.35)	-	-
**Hispanic/Latino ethnicity**					
No	-	-	ref	-	-
Yes	-	-	0.35** (0.19–0.67)	-	-
Unknown	-	-	0.49 (0.14–1.67)	-	-
**Years of education**			0.98 (0.96–1.01)		
More than 12 years of education	-	-	-	-	-
12 years or less	-	ref	-	ref	ref
More than 12 years	-	0.86 (0.73–1.02)	-	0.93 (0.76–1.14)	0.96 (0.78–1.17)
**Type of residence**					
Single- or multi-family private residence (apartment, condo, house)	-	-	ref	ref	ref
Retirement community or independent group living	-	-	1.31** (1.07–1.60)	1.44*** (1.19–1.75)	1.36** (1.12–1.65)
Institutionalized	-	-	2.49*** (1.72–3.60)	3.1*** (2.22–4.33)	3.11*** (2.28–4.25)
***APOE* status**					
No ε4 allele	-	-	ref	ref	ref
One or two copies of ε4 allele	-	-	1.09 (0.91–1.29)	1.11 (0.94–1.30)	1.05 (0.89–1.25)
**Presence or history of CAD**					
No	-	-	ref	-	-
Yes	-	-	1.93*** (1.50–2.49)	-	-
**Presence or history of CVD**					
No	-	-	ref	-	-
Yes	-	-	1.32 (0.99–1.75)	-	-
**Currently smoking**					
No	-	-	ref	-	-
Yes	-	-	2.06*** (1.37–3.10)	-	-
**Alcohol abuse**					
Absent	-	-	ref	-	-
Recent/Active	-	-	1.4 (0.63–3.11)	-	-
Remote/Inactive	-	-	1.66** (1.15–2.38)	-	-
**Disease severity by age at start of stage**					
MCI due to AD by age at index visit	-	-	-	-	0.99 (0.96–1.03)
Mild AD by age at index visit	-	-	-	-	0.93** (0.89–0.98)
Moderate AD by age at index visit	-	-	-	-	0.9*** (0.87–0.93)
Severe AD by age at index visit	-	-	-	-	0.9*** (0.87–0.94)
**Number of observations per stage**					
CN	11,458	11,395	8859	8971	8971
MCI due to AD	937	932	497	777	777
Mild AD dementia	522	521	367	456	456
Moderate AD dementia	457	456	276	395	395
Severe AD dementia	85	85	53	69	69

*AD* Alzheimer's disease, *APOE* Apolipoprotein E, *BMI* Body mass index, *CAD* Coronary artery disease, *CI* Confidence interval, *CN* Cognitively normal, *CVD* Cerebrovascular disease, *MCI* Mild cognitive impairment, *ref* reference category

^*^p < 0.05; **p < 0.01, ***p < 0.001

**Fig. 2:**
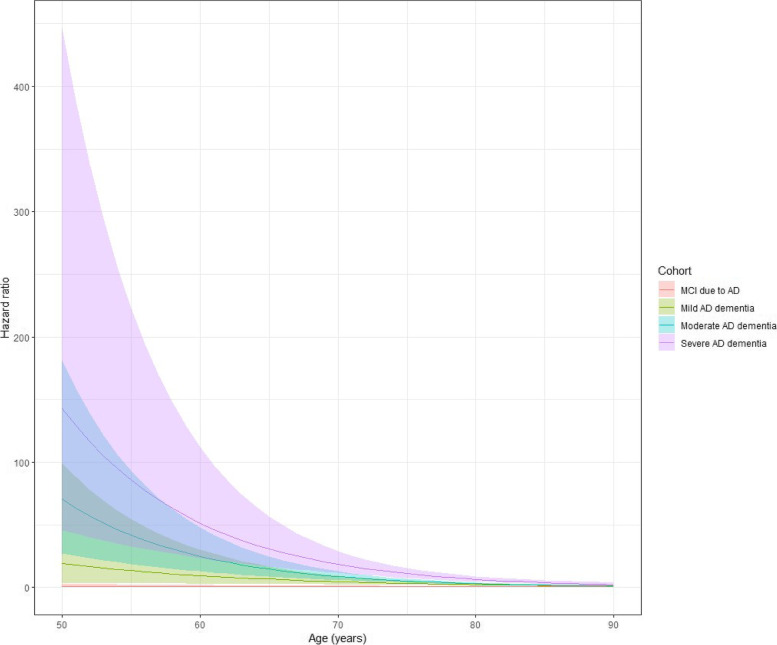
Hazard ratios (filled lines) with 95% confidence intervals (dashed lines) of risk of death compared with cognitively normal participants by disease stage (panels) and age (x-axis). *AD Alzheimer’s disease*

Of note, there was a statistically significant interaction between age and disease severity, suggesting that the relative effect on mortality from increasing disease severity is higher in younger participants (Fig. 2). For instance, the HRs (95% CI) for mortality at 65 years of age were 6.7 (2.7–16.9), 14.8 (8.8–24.8), and 30.1 (16.7–56.8) for mild, moderate, and severe AD, respectively; at 80 years of age, the corresponding HRs (95% CI) were 2.4 (1.7–3.3), 3.1 (2.4–3.9), and 6.6 (4.8–9.1), respectively (Supplementary Table 4).

### Sensitivity analyses

The two sensitivity analyses on the exploratory model 3, i.e., removing comorbidity predictors and limiting data to pre-COVID-19 lockdown, only showed minor impacts on the results (Supplementary Table 5). Participants with MCI due to AD with a positive AD biomarker (*n* = 47) were similar to the overall MCI due to AD cohort (Supplementary Table 6), but there were too few participants for a separate analysis. Back-transitions to less-severe stages did occur, but in a minority of cases (Supplementary Table 2). Participants lost to follow-up were somewhat younger, more often females, and, on average, had fewer symptoms (Supplementary Table 9). This may be expected because these factors reduce the risk of death and, with regard to age and symptoms, also progression, which are competing risks of loss to follow-up.

The alternative classification model of disease severity based on CDR-SB resulted in slightly different estimates, but did not change the direction of any effects and resulted in marginal changes to the significance of some predictors (Supplementary Tables 7 and 4). Overall, the relative mortality risks in AD dementia stages were somewhat higher with the CDR-SB model compared with the multi-domain model. Notably, the number of participants with CDR-SB scores available (*n* = 12,448) was lower than those included in the multi-domain model (*n* = 13,459), and especially low in the AD dementia group.

## Discussion

### Main findings

In this study, we show that all-cause mortality increases with the severity of symptoms in AD dementia, including when controlling for other predictors of mortality. We also show that this relative increase in the risk for death is higher in younger people. This is supported by previous studies [12] but to our knowledge has not been considered in earlier reports on relative risks. It implies that we can expect increased longevity from AD prevention interventions, and importantly, that this potential benefit is greater in relatively younger people. For example, according to our estimates, an 80-year-old person with severe AD dementia has a sevenfold risk of dying compared with an 80-year-old CN person, whereas the corresponding risk increase for a 65-year-old person with severe AD dementia is 31 times higher (albeit with a wide CI ranging from 16.7 to 56.8, suggesting inadequate power and lower estimation precision). The relatively lower impact on mortality from AD in advanced ages is likely due to competing causes of death. It should be noted that specific causes of death are not captured in the NACC UDS and are therefore not considered in this analysis.

We found no increase in mortality associated with MCI due to AD after controlling for confounding factors and, importantly, disease progression over time. People in the early stages of AD should indeed be expected to have a shorter survival than CN persons, because most will progress to AD dementia over time [46] and AD dementia clearly causes increased mortality. This has been demonstrated by previous studies albeit in persons with MCI due to any cause [47–49]; however, our analysis further suggests that there is no significant increase in mortality for people while remaining in the MCI due to AD stage. This does not mean that they will not have an increased risk of dying later, which, again, is consistent with previous studies. In our Cox proportional-hazards models, participants with MCI due to AD who progressed to AD dementia were censored from this particular cohort at the time of progression. However, we cannot assume that these censored participants would have the same mortality risk going forward as compared with those remaining in the MCI due to AD stage. The Kaplan–Meier estimates on unadjusted and non-censored data were also consistent with this interpretation. The higher death rate in people with MCI due to AD compared with CN people may be explained by more advanced age and relatively more men in the MCI due to AD stage, in addition to this population having a higher rate of progression to AD dementia, which is associated with higher mortality. However, there is a possibility that our methods prevented us from finding an actual difference in mortality, as well as in MCI due to AD. Our eligibility criteria were somewhat more restrictive for AD cohorts compared with CN participants, as we excluded participants with a record of non-AD etiologic diagnosis potentially causing cognitive impairment before or at index. This may have resulted in underestimation of the relative mortality in MCI due to AD compared with CN participants.

Overall, our findings on increasing mortality with increasing severity of dementia are consistent with previous studies [13, 14, 24–26], albeit with varying effect sizes, probably largely explained by differences in the source populations, definitions of disease stages, and methodologies. For example, the HRs reported by Andersen et al. [26] from a Danish cohort of participants with prevalent and incident dementia were approximately 3, 4, and 10 for mild, moderate, and severe dementia, respectively, defined by CDR and after adjusting for age and sex. These estimates were quite close to the estimates from our sensitivity analysis using CDR-SB (model 2 resulted in HRs 2, 4, and 9 for mild, moderate, and severe AD dementia, respectively). Also, consistent with previous studies, we found that male sex [13–17, 47] and residential or institutionalized care setting are important predictors of increased mortality [24]. Other significant factors that emerged included presence or history of CAD, currently smoking, alcohol abuse, being overweight, Asian race, and Hispanic ethnicity (levels of education and APOE status were not significant factors). Moreover, we should expect collinearity between several variables, which may imply that the effect of some predictors of mortality is nullified when controlling for others; however, we employed methodology (i.e., Lasso) to try to account for such a limitation.

This study did not and could not assess the causality of AD progression on mortality. This potentially causal effect needs to be tested in experimental studies, long enough to show an impact of AD prevention on the mortality outcome. Although many studies have shown that AD is associated with premature death, this association does not necessarily need to be constant when modifying the progression of the disease. In other words, delaying disease progression, as measured by severity of symptoms or AD biomarkers, does not necessarily imply increased longevity, although this is highly likely.

### Strengths

One strength of this study was the long follow-up (up to 15 years) on a large (*n* = 12,414) and well-characterized study population, encompassing all clinical stages of AD and CN participants. This enabled us to consider incident cases of AD and study them from the start of any particular clinical disease stage to progression or death. Another strength was the availability of annual assessments across all relevant symptom domains, including cognition, function, and behavior, which allowed us to classify participants into disease stages according to a comprehensive multi-domain disease model, and not just by MMSE scores. Furthermore, the availability of data on etiology (albeit limited biomarker data), comorbidities, and other characteristics allowed us to control for potential confounding factors for mortality. The resulting adjusted models increased the generalizability of our findings to populations that differ from the NACC UDS, e.g., in terms of age or sex.

### Limitations

Our study had several limitations. Firstly, the NACC UDS is a volunteer-based case series data set and is not considered a representative sample of the general US population and therefore includes limited variation between some variables. For instance, participants are more highly educated and non-Hispanic White persons are over-represented compared with the general US population limiting the study’s ability to draw conclusions about mortality risks in different racial and ethnic groups, and thus in the overall US population [50]. Non-Hispanic White persons represent approximately 71% of all participants in the NACC UDS [51], compared with 59% of the general US population [52]. The NACC UDS population is also more highly educated and women are slightly over-represented (57% in the NACC UDS vs 51% of the general US population). Comparison of our data to life tables suggests that our cohort may have longer average life expectancy than the general US population. As an example, a subset of CN participants (414 women and 287 men) aged 85 years and above had a median survival of 9.4 and 7.2 years, respectively, which is higher than the expected survival in the US of 85 years of age: median survival 7.1 years for women and 6.0 years for men [53]. The difference may be explained by the inclusion of people with dementia in the latter estimate, which should result in lower overall survival. However, the selection of participants into the NACC UDS may have caused us to underestimate the mortality in CN participants and therefore overestimate the relative risk of death in AD.

Secondly, there is a risk of underreporting of mortality because the NACC UDS relied on ADRCs to report deaths and data were therefore only available for actively followed participants. This problem was mitigated by censoring participants without a recent follow-up, but this may have instead increased the risk of informative censoring, i.e., if participants with a higher/lower mortality have a higher risk of being lost to follow-up. This may have biased our HRs if censoring was more common in either of the cohorts. Examining the attrition rates (Supplementary Table 1), we noticed some variation across cohorts. However, censoring due to loss to follow-up was most common in the CN and MCI due to AD cohorts (as well as moderate AD dementia), in contrast to what would be expected if increased disease severity increases the risk of discontinuation. In fact, there was no consistent association between stage and percentage lost to follow-up. Furthermore, participants who were lost to follow-up were similar to those who were not, and, if anything, were slightly younger, more often female, and with fewer symptoms overall. Both these findings may suggest that discontinuation is not primarily associated with increased disease severity or risk of death, and therefore a lower risk of informative censoring. However, acknowledging there is still a risk of bias due to informative censoring in our analysis; this most likely should have caused us to underestimate the relative risk of death in AD, at least if relatively healthy participants in each cohort had a higher likelihood of continuing.

Thirdly, there is a risk of misclassification bias, both in terms of etiology of symptoms and staging of disease. Biomarker data were only available in a small subset of participants and could therefore not be used to confirm AD etiology in participants with a clinical AD diagnosis. This may have caused us to misclassify both AD and non-AD participants across all cohorts; especially in MCI due to AD where symptoms are subtle. Encouragingly, in a previous NACC study, investigators reported that 438 (83.3%) of 526 participants with clinically probable AD actually met neuropathologic criteria for AD [54]. Also, baseline characteristics of participants with biomarker-confirmed MCI due to AD were similar to those of participants in the MCI due to AD cohort (Supplementary Table 6). Similarly, the staging of disease severity is dependent on the accuracy of individual tests and the scoring algorithm to assign participants to different stages. Cognitive measurements are imprecise and may fluctuate over time [36]. This is seen in back-transitions to less-severe stages (Supplementary Table 2). We chose to disregard back-transitions as they are likely temporary in most cases. In a previous study, 38% of MCI participants reverted to CN, but 65% subsequently developed MCI or dementia, suggesting that a diagnosis of MCI at any time, even among participants who revert to CN, has prognostic value for AD dementia [37]. Our sensitivity analysis showed that a different algorithm, classifying people into severity stages by CDR-SB, had some impact on the HRs (Supplementary Table 7). There is no prevailing standard for the definition of disease stages, and it is therefore important to make sure that disease stages are defined consistently across combined data sets and models. Other factors that may have caused misclassification include changing criteria for diagnosis across the NACC UDS versions (National Institute of Neurological and Communicative Diseases and Stroke/Alzheimer's Disease and Related Disorders Association [NINCDS/ADRDA] in version 1 [55] and version 2 [56] and NIA – Alzheimer's Association [NIA-AA] in version 3 [57]), as well as potential reporting biases (e.g., via participant health history) and missingness in variables used to determine comorbidity.

A fourth noteworthy, but probably marginal, limitation is related to the lack of precision in the timing of the data records. Death was recorded by year and month in the NACC UDS, with the death date set to the last day of the month to avoid negative follow-up time. This should have resulted in an average overestimation of survival across all groups of about half a month. Similarly, events of progression were not continuously recorded (as per other AD studies, to our knowledge), which implies that participants may have progressed earlier than at the visit when progression was assessed. Neither of these limitations should have had any meaningful impact on our results.

Taken together, these limitations should cancel each other out to some degree but there remains a risk of us over- or underestimating the true increase in the risk of death in AD.

## Conclusions

We conclude three important findings from this study. Firstly, our data show a relative increase in the risk of death in people in more severe stages of AD. Secondly, this effect is higher in relatively younger persons. Thirdly, our data suggest no increased mortality in persons with MCI due to AD while remaining in this disease stage.

These findings have two major implications. They show that there is a potential benefit of decreased mortality if preventing or delaying the progression of AD is successful, and this benefit is greatest in relatively younger persons and as long as they remain in the MCI due to AD stage. Also, they show the importance of considering both age and disease stage, as well as their interaction, when evaluating health campaigns, prevention strategies, and treatment interventions. The HRs from our Cox proportional-hazards models can be used for this purpose.

In future research, our results should be replicated in other samples more generalizable to the general US population as well as other populations around the world, preferably with linkage to mortality/vital records for more complete reporting of death, and more extensive biomarker-based characterization of persons with AD. Ultimately, clinical trials are necessary to more fully assess the potential causal effect of preventing or delaying the progression of AD on mortality.

### Supplementary Information

**Additional file 1.**

**Additional file 2.**

## Data Availability

Individual-level data were sourced from the Uniform Data Set of the National Alzheimer’s Coordinating Center. Information about the data used in this study, including detailed descriptions and the process for obtaining them, is available at https://naccdata.org/.

## Abbreviations

AD: Alzheimer’s disease
ADRC: Alzheimer’s Disease Research Center
APOE: Apolipoprotein E
BMI: Body mass index
CAD: Coronary artery disease
CDR-GS: Clinical Dementia Rating – Global Score
CDR-SB: Clinical Dementia Rating – Sum of Boxes
CI: Confidence interval
CN: Cognitively normal
FAQ: Functional Activities Questionnaire
HR: Hazard ratio
MCI: Mild cognitive impairment
MMSE: Mini-Mental State Examination
MoCA: Montreal Cognitive Assessment
NACC: National Alzheimer’s Coordinating Center
NIA: National Institute on Aging
NIA-AA: National Institute on Aging – Alzheimer's Association
NINCDS/ADRDA: National Institute of Neurological and Communicative Diseases and Stroke/Alzheimer's Disease and Related Disorders Association
NPI-Q: Neuropsychiatric Inventory Questionnaire
UDS: Uniform Data Set
