# Supplementary material for: Disease severity and mortality in Alzheimer's disease: an analysis using the U.S. National Alzheimer’s Coordinating Center Uniform Data Set
Source: BMC Neurol. 2023 Aug 14;23:302. doi: 10.1186/s12883-023-03353-w (PMC10424331; doi:10.1186/s12883-023-03353-w)
Supplement: Supplementary file 1 — Additional file 1. [file 12883_2023_3353_MOESM1_ESM.docx]

**Supplement**

**Eligibility criteria and cohorts**

Cognitively normal (CN) participants had a clinical diagnosis of normal cognition at index (National Alzheimer’s Coordinating Center cognitive status at Uniform Data Set visit [NACCUDSD] = 1) confirmed by Clinical Dementia Rating (CDR) Dementia Staging Instrument® scores at index (Clinical Dementia Rating–Global and Sum of Boxes [CDRGLOB and CDRSUM] = 0), and no prior record of impairment at any pre-index visit (NACCUDSD > 1, CDRGLOB > 0 or CDRSUM > 0).

Participants with mild cognitive impairment (MCI) due to Alzheimer’s disease (AD) had a clinical diagnosis of amnestic or non-amnestic MCI (NACCUDSD = 3) and CDRGLOB ≤ 0.5 at index, and no prior record of more severe impairment (NACCUDSD > 2 or CDRGLOB > 0.5). To limit to incident participants, they were further required to have a pre-index visit with normal cognition (NACCUDSD = 1), or impaired not MCI (NACCUDSD = 2), and CDRGLOB ≤ 0.5 within 15 months before the index date. They were also required to have a record of AD etiology (NACCALZD = 1) at index or after, and no record of non-AD etiologic diagnosis potentially causing cognitive impairment before or at index, with the exception of anxiety (ANXIET) and depression (DEP), because these have been suggested to be possible first signs of AD [1, 2].

Participants with AD dementia had a clinical diagnosis of dementia (NACCUDSD = 4), CDRGLOB ≥ 0.5 and Mini-Mental State Examination (MMSE) ≤ 26 at index, and no prior record of such impairment (NACCUDSD > 3). They were required to have a pre-index visit with normal cognition, impaired not MCI or MCI (NACCUDSD ≤ 3), and CDRGLOB ≤ 0.5 within 15 months before the index date. They were also required to have a presumptive primary etiologic diagnosis of AD (NACCALZD = 1 and NACCALZP = 1) at index, and no record of non-AD etiologic diagnosis potentially causing cognitive impairment before or at index, with the exception of ANXIET and DEP. Finally, in order to enable further dementia staging, participants with AD dementia were required to have complete MMSE or Montreal Cognitive Assessment (MoCA) scores, complete Neuropsychiatric Inventory Questionnaire (NPI-Q) scores, and at least five completed tasks for the Functional Activities Questionnaire (FAQ) scale at index.

The authors of this study have interpreted the NACCALZD variable as primarily determined by clinical assessment, although it may be supported by biomarker confirmation in later observations. The NACC Data Dictionary says: *“It is important to note that the criteria for an etiologic diagnosis of Alzheimer’s disease is different in versions 1-2 and 3: in v1.2 and v2, the NINCDS/ADRDA criteria were applied and in v3 the NIA-AA criteria for AD dementia are applied.”*

Non-AD etiologies were identified from other etiologic flags in the D1 variable group (clinician presumptive etiologic diagnosis of the cognitive disorder) of the NACC data; namely, NACCLBDE, MSA, PSP, CORT, FTD, FTLDMO, FTLDNOS, PPAPH, CVD, VASC, STROKE, ESSTREM, DOWNS, HUNT, PRION, BRNINJ, HYCEPH, EPILEP, NEOP, HIV, OTHCOG, DEP, BIPOLDX, SCHIZOP, ANXIET, DELIR, PTSDDX, OTHPSY, ALCDEM, IMPSUB, DYSILL, MEDS. Individuals whose cognitive disorder had undetermined etiology (DEMUN = 1) were retained in the sample.

If participants had a non-AD etiologic diagnosis post-index, they were retained in the sample and considered to have mixed dementia or dementia due to multiple causes; these are likely to be included in the label population even if they were excluded from the clinical trial.

Participants with AD dementia were further classified into one of three disease severity stages (mild, moderate, and severe), according to a previously published model using scores on cognitive, behavioral, and functional symptom domains as measured by the MMSE, NPI-Q, and FAQ scales [3]. By this model, people with AD dementia are classified as mild AD dementia if they were mild in cognition (21 ≤ MMSE ≤ 26) while neither severe in behavior (at least one NPI-Q item = 3) nor function (FAQ total ≥ 24); moderate AD dementia if moderate in cognition (10 ≤ MMSE ≤ 20) while not severe in both behavior or function, or mild in cognition while severe in either behavior or function; and severe AD dementia if severe in cognition (MMSE ≤9) or moderate in cognition while severe in both behavior and function. Because MMSE has been replaced by MoCA in NACC Uniform Data Set (UDS) since March 2015, these scores were mapped using a published conversion algorithm [4].

Behavior: the NPI-Q was developed to assess the frequency, severity, and caregiver distress of 12 different neuropsychiatric disturbances common in dementia. Only questions about presence or absence and the severity rating of each disturbance from the NPI are contained within the UDS; the severity score ranges from 0 to 36, with the highest score representing more severe neuropsychiatric symptoms. There is no direct NACC variable for the severity score, thus it was computed by summing up the NPI-Q components using the following variables: DELSEV, HALLSEV, AGITSEV, DEPDSEV, ANXSEV, ELATSEV, APASEV, DISNSEV, IRRSEV, MOTSEV, NITESEV, APPSEV. When the value of any of these variables = 8 (not applicable, no symptoms reported), the severity rating for that symptom was taken to be zero. Only participants having valid scores (i.e., with variable value = 1, 2, 3, or 8) on all items were considered for data analyses using the NPI-Q.

Function: measured over time by the sum FAQ score, ranging from 0 to 30, with the highest score representing the lowest function (no direct NACC variable, it was computed from the 10 following variables: BILLS, TAXES, SHOPPING, GAMES, STOVE, MEALPREP, EVENTS, PAYATTN, REMDATES, TRAVEL). Each item is rated on a four-point scale (0 = normal; 1 = has difficulty by does by self; 2 = requires assistance; 3 = dependent). In the UDS, “not applicable” (i.e., never did) is an allowable response if the participant never did the activity. Even one “not applicable” response precludes calculation of a FAQ score. In this analysis, we replaced “not applicable” responses with the mean of completed FAQ items at each participant visit, if at least half of the items were completed [5].

**Follow-up details**

Participants were followed until the event of death, progression to a more severe disease stage, or when censored (either at the end of the study period, or due to discontinuation, or because information to determine disease stage was missing). Participants were assumed to have discontinued either if recorded as such (NACCACTV = 0 or 2, with recorded date or, if missing, at last visit) or at 15 months after the last recorded visit. Participants were censored due to missing information at the last visit before a gap of 36 months or more between two consecutive visits, with the exception of participants with severe AD dementia who were assumed to remain in this disease stage.

If, at post-index visits, MMSE/MOCA and/or NPI-Q scores were missing, and/or there were < 5 completed tasks for the FAQ scale, the patient was assumed to be in the same stage as at the previous visit where complete information was available. If there was a lag of > 36 months between visits where complete information was available, the stage was not imputed unless the patient was in the severe AD stage at last visit.

**Predictors and covariates**

Presence or history of coronary artery disease (CAD) and presence or history of cerebrovascular disease (CVD) were included as potential confounders. A binary CAD variable was created using the variables MYOINF, CVHATT, CVANGINA, and ANGINA. A binary CVD variable was created using the variables CBSTROKE, CBTIA, HXSTROKE, PREVSTK. Presence or history of coronary artery disease and presence or history of cerebrovascular disease were based on all records prior to and including index record. CAD and CVD were included as covariates to represent cerebro- and cardiovascular disease, rather than underlying risk-factors such as hypertension and diabetes, because the former are more common primary causes of death [6].

Type of residence was determined using the RESIDENC variable, with RESIDENC = 3 and 4 classified as institutionalized.

**Statistical analysis**

Prior to fitting Lasso regression, baseline predictors with zero or near-zero variance were removed (applied to TOBAC30, ALCOHOL, and CAD). Next highly correlated (> 0.75) baseline predictors were identified and, per clinical input, removed (none were removed). The remaining baseline predictors were standardized so that their mean and variance are 0 and 1, respectively. Lasso selects the predictors through a shrinking parameter (lambda). The optimal lambda was obtained via fivefold nested cross-validation (CV). Nested CV uses a series of training/validation splits of the training data avoiding correlation between the folds. The test set was used to assess the estimated model parameters. The remaining parameters in the final Lasso model were used to assess the strength of the association (hazard ratio and 95% confidence intervals) between baseline predictors and mortality using Cox proportional-hazards regression, based on complete case analysis (i.e., missing data were not imputed).

**Sensitivity analyses**

AD Biomarker Positive was based on all available records.

**Supplementary Tables**

**Supplementary Table 1**. Follow-up and attrition from study cohorts (some participants included in several cohorts if progressing). **Supplementary Table 2**. Observed back-transitions in NACC UDS. **Supplementary Table 3**. Identification of cohorts in NACC UDS according to eligibility criteria (some participants included in several cohorts if progressing). **Supplementary Table 4**. Predicted hazard ratios for different age groups, based on model 5**. Supplementary Table 5**. Sensitivity analysis on relative risks of death estimated by Cox proportional-hazards models. **Supplementary Table 6.** Participant characteristics comparing MCI due to AD cohort with subset with positive AD biomarker. **Supplementary Table 7.** Sensitivity analysis of relative risks of death estimated by Cox proportional-hazards models (classifying disease severity by CDR-SB). **Supplementary Table 8.** Participant characteristics of complete cases contributing to each model (including multiple observations from some participants included in several cohorts if progressing). **Supplementary Table 9.** Participant Characteristics Comparing Those Lost to Follow-Up and Those That Died, Progressed or Continued Until Data Cut (Including Multiple Observations from Some Participants Included in Several Cohorts if Progressing).

**NACC variable definitions (for more information, please consult the NACC UDS Researchers Data Dictionary (https://files.alz.washington.edu/documentation/uds3-rdd.pdf)**

NACCACTV: Follow-up status at the Alzheimer’s Disease Center (ADC). 0 = died, discontinued, lost to follow up; 1 = annual follow-up (no discontinuation/loss to follow-up or minimal contact; 2 = minimal contact with Center, no annual follow-up.

RESIDENC: Type of residence. 1 = single- or multi-family private residence (apartment, condo, house); 2 = retirement community or independent group living; 3 = assisted living, adult family home, or boarding home; 4 = skilled nursing facility, nursing home, hospital, or hospice; 9= other or unknown

CVHATT: Heart attack/cardiac arrest history (mainly subject and co-participant report)

CVANGINA: Angina history (mainly subject and co-participant report)

CBSTROKE: Stroke history (mainly subject and co-participant report)

CBTIA: Transient ischemic attack history (mainly subject and co-participant report)

HXSTROKE: History of stroke

CDRSUM: Standard CDR® sum of boxes

CDRGLOB: Global CDR®

NACCUDSD: Cognitive status at UDS visit. 1 = normal cognition; 2 = impaired not MCI; 3 = MCI; 4 = Dementia

NACCALZD: Presumptive etiologic diagnosis of the cognitive disorder – Alzheimer’s disease. 0 = no (assumed assessed and found not present); 1 = yes; 8 = no cognitive impairment

NACCALZP: Primary, contributing, or non-contributing cause of observed cognitive impairment – Alzheimer’s disease. 1 = primary; 2 = contributing; 3 = non-contributing; 7 = cognitively impaired but not AD diagnosis; 8 = diagnosis of normal cognition

NACCLBDE: Lewy body disease

MSA: Multiple system atrophy

PSP: Primary supranuclear palsy

CORT: Corticobasal degeneration

FTLDMO: Frontotemporal lobar degeneration (FTLD) with motor neuron disease

FTLDNOS: FTLD not otherwise specified

FTD: Presence of behavioral frontotemporal dementia

PPAPH: Primary progressive aphasia

CVD: Vascular brain injury

PREVSTK: Previous symptomatic stroke

VASC: Probable vascular dementia

STROKE: Stroke

ESSTREM: Essential tremor

DOWNS: Down syndrome

HUNT: Huntington’s disease

PRION: Prion disease

BRNINJ: Traumatic brain injury

HYCEPH: Normal-pressure hydrocephalus

EPILEP: Epilepsy

NEOP: Central nervous system (CNS) neoplasm

HIV: Human immunodeficiency virus

OTHCOG: Other neurological, genetic, or infectious condition

DEP: Depression

BIPOLDX: Bipolar disorder

SCHIZOP: Schizophrenia or other psychosis

ANXIET: Anxiety

DELIR: Delirium

PTSDDX: Post-traumatic stress disorder

OTHPSY: Other psychiatric disease

ALCDEM: Cognitive impairment due to alcohol abuse

IMPSUB : Cognitive impairment due to other substance abuse

DYSILL: Cognitive impairment due to systemic disease/medical illness

MEDS: Cognitive impairment due to medications

DEMUN: Undetermined etiology

MYOINF: Myocardial infarct present within the past 12 months (clinician-assessed)

ANGINA: Angina present (clinician-assessed)

**References**

[1] Dafsari FS, Jessen F. Depression-an underrecognized target for prevention of dementia in Alzheimer's disease. Transl Psychiatry. 2020;10:160.

[2] Hanseeuw BJ, Jonas V, Jackson J, Betensky RA, Rentz DM, Johnson KA, et al. Association of anxiety with subcortical amyloidosis in cognitively normal older adults. Mol Psychiatry. 2020;25:2599-607.

[3] Green C, Zhang S. Predicting the progression of Alzheimer's disease dementia: A multidomain health policy model. Alzheimers Dement. 2016;12:776-85.

[4] Trzepacz PT, Hochstetler H, Wang S, Walker B, Saykin AJ, Alzheimer's Disease Neuroimaging I. Relationship between the Montreal Cognitive Assessment and Mini-mental State Examination for assessment of mild cognitive impairment in older adults. BMC Geriatr. 2015;15:107.

[5] Gill DP, Hubbard RA, Koepsell TD, Borrie MJ, Petrella RJ, Knopman DS, et al. Differences in rate of functional decline across three dementia types. Alzheimers Dement. 2013;9:S63-71.

[6] Murphy SL, Kochanek KD, Xu J, Arias E. Mortality in the United States, 2020. NCHS Data Brief. 2021:1-8.
