# Supplementary material for: Disease severity and mortality in Alzheimer's disease: an analysis using the U.S. National Alzheimer’s Coordinating Center Uniform Data Set
Source: BMC Neurol. 2023 Aug 14;23:302. doi: 10.1186/s12883-023-03353-w (PMC10424331; doi:10.1186/s12883-023-03353-w)
Supplement: Supplementary file 2 — Additional file 2. [file 12883_2023_3353_MOESM2_ESM.docx]

**Supplementary Table 1** Follow-up and attrition from study cohorts (some participants included in several cohorts if progressing)

|  | CN  *N* = 11,458 | MCI due to AD  *N* = 937 | Mild AD  *N* = 522 | Moderate AD  *N* = 457 | Severe AD  *N* = 85 | Total  *N* = 13,459 |
| --- | --- | --- | --- | --- | --- | --- |
| Died, progressed, or censored at data cut, *n* (%) | **5617 (0.5)** | **529 (0.6)** | **331 (0.6)** | **225 (0.5)** | **64 (0.8)** | **6766 (0.5)** |
| Died | 585 (5.1) | 102 (10.9) | 51 (9.8) | 118 (25.8) | 61 (71.8) | 917 (6.8) |
| Discontinued due to disease progression | 2931 (25.6) | 273 (29.1) | 254 (48.7) | 64 (14.0) | 0 (0) | 3522 (26.2) |
| Under follow-up till data cut off (March 1, 2021) | 2101 (18.3) | 154 (16.4) | 26 (5.0) | 43 (9.4) | 3 (3.5) | 2327 (17.3) |
| Censored due to loss to follow-up or discontinue due to missing information, *n* (%) | **5841 (0.5)** | **408 (0.4)** | **191 (0.4)** | **232 (0.5)** | **21 (0.2)** | **6693 (0.5)** |
| Discontinued as recorded from annual follow-up | 3349 (29.2) | 236 (25.2) | 143 (27.4) | 168 (36.8) | 14 (16.5) | 3910 (29.1) |
| Under annual follow-up as recorded in the NACC and discontinued due to lack of visit for >15 months | 2045 (17.8) | 150 (16.0) | 30 (5.7) | 38 (8.3) | 7 (8.2) | 2270 (16.9) |
| Discontinued due to >36 months gap between subsequent follow-up (not applicable to severe AD dementia) | 447 (3.9) | 22 (2.3) | 11 (2.1) | 9 (2.0) | 0 (0) | 489 (3.6) |
| Discontinued due to missing stage for >36 months (AD patients only) | 0 (0) | 0 (0) | 7 (1.3) | 17 (3.7) | 0 (0) | 24 (0.2) |
| Follow-up duration (years) |  |  |  |  |  |  |
| Mean (SD) | 4.44 (3.47) | 3.65 (2.84) | 2.18 (1.52) | 2.79 (1.81) | 2.79 (1.93) | 4.23 (3.37) |
| Median (Q1, Q3) | 3.30 (1.73, 6.18) | 2.96 (1.38, 4.84) | 1.86 (1.07, 2.92) | 2.49 (1.29, 3.73) | 2.68 (1.23, 3.88) | 3.13 (1.55, 5.82) |
| Min, Max | 0.00274, 15.5 | 0.0164, 14.1 | 0.00548, 12.3 | 0.0219, 12.1 | 0.0164, 9.54 | 0.00274, 15.5 |

AD, Alzheimer's disease; CN, cognitively normal; MCI, mild cognitive impairment; NACC, National Alzheimer's Coordinating Center; Q1, first quartile; Q3, third quartile; SD, standard deviation.

**Supplementary Table 2** Observed back-transitions in NACC UDS

|  | Total | Reverse to cognitively normal | Reverse to MCI due to AD | Reverse to mild AD dementia | Reverse to moderate AD dementia |
| --- | --- | --- | --- | --- | --- |
| Cognitively normal | 11,458 | - | - | - | - |
| MCI due to AD | 937 | 185 | - | - | - |
| Mild AD dementia | 522 | 3 | 24 | - | - |
| Moderate AD dementia | 457 | - | 8 | 76 | - |
| Severe AD dementia | 86 | - | - | - | 12 |

AD, Alzheimer's disease; MCI, mild cognitive impairment; NACC, National Alzheimer's Coordinating Center; UDS, Uniform Data Set.

**Supplementary Table 3** Identification of cohorts in NACC UDS according to eligibility criteria (some participants included in several cohorts if progressing)

| Eligibility criteria by cohort (base case analysis) | *n* |
| --- | --- |
| Cognitively normal |  |
| Total patients with visits at age >= 50 years | 33,874 |
| At index date, clinical diagnosis of normal cognition and CDR-GS and CDR-SB equal to 0 | 13,383 |
| Exclude patients with NACCUDSD > 1 or CDR-GS >0 or CDR-SB >0 at any visit prior to the index date | 12,282 |
| Exclude patients with follow-up duration <= 0 day | 11,458 |
| MCI due to AD |  |
| Total patients with visits at age >= 50 years | 33,874 |
| At index date, clinical diagnosis of amnestic or non-amnestic MCI and CDR-GS <= 0.5, and age >= 50 years | 2740 |
| Pre-index visit with normal cognition or impaired, not MCI and CDR-GS <= 0.5 within 15 months before the index date | 1815 |
| At least one visit with a record for AD etiology on or after index date | 1285 |
| Exclude patients with CDR-GS > 0.5 or NACCUDSD > 2 at any visit prior to the index date | 1269 |
| Exclude patients with non-AD etiologic diagnosis potentially causing cognitive impairment before or at index date, with the exception of anxiety and depression | 951 |
| Exclude patients with follow-up duration <= 0 day | 937 |
| AD dementia |  |
| Total patients with visits at age >= 50 years | 33,874 |
| At index date, clinical diagnosis of dementia, presumptive primary etiologic diagnosis of AD, CDR-GS >= 0.5, and MMSE <= 26, age >= 50 years | 1663 |
| Pre-index visit with normal cognition, impaired not MCI or MCI, and CDR-GS <= 0.5 within 15 months before the index date | 1145 |
| Complete MMSE/MOCA scores and NPI-Q scores and at least five completed tasks for the FAQ scale at index | 1082 |
| Exclude patients with NACCUDSD > 3 at any visit prior to the index date | 1082 |
| Exclude patients with non-AD etiologic diagnosis potentially causing cognitive impairment before or at index date, with the exception of anxiety and depression | 783 |
| Exclude patients with follow-up duration <= 0 day | 745 |
|  |  |
| Sensitivity analysis classifying disease severity by CDR-SB | ***n*** |
| Cognitively normal |  |
| Total patients with visits at age >= 50 years | 33,874 |
| At index date, clinical diagnosis of normal cognition and CDR-SB equal to 0 | 13,383 |
| Exclude patients with NACCUDSD > 1 or CDR-SB > 0 at any visit prior to the index date | 12,282 |
| Exclude patients with follow-up duration <= 0 day | 11,458 |
| MCI due to AD |  |
| Total patients with visits at age >= 50 years | 33,874 |
| At index date, clinical diagnosis of amnestic or non-amnestic MCI (NACCUDSD = 3) and 0.5 <= CDR-SB <= 4.0,  and age >= 50 years | 2335 |
| Pre-index visit with normal cognition or impaired, not MCI, and CDR-SB < 0.5 within 15 months before the index date | 1184 |
| At least one visit with a record for AD etiology on or after index date | 870 |
| Exclude patients with CDR-SB >= 0.5 or NACCUDSD > 2 at any visit prior to the index date | 525 |
| Exclude patients with non-AD etiologic diagnosis potentially causing cognitive impairment before or at index date, with the exception of anxiety and depression | 419 |
| Exclude patients with follow-up duration <= 0 day | 415 |
| AD dementia |  |
| Total patients with visits at age >= 50 years | 33,874 |
| At index date, clinical diagnosis of dementia, presumptive primary etiologic diagnosis of AD, CDR-SB >= 4.5, age >= 50 years | 1121 |
| Pre-index visit with normal cognition, impaired not MCI or MCI, and CDR-SB <= 4.0 within 15 months before the index date | 653 |
| Complete CDR-SB score at index | 653 |
| Exclude patients with NACCUDSD > 3 or CDR-SB >= 4.5 at any visit prior to the index date | 653 |
| Exclude patients with non-AD etiologic diagnosis potentially causing cognitive impairment before or at index date, with the exception of anxiety and depression | 429 |
| Exclude patients with follow-up duration <= 0 day | 414 |

AD, Alzheimer's disease; CDR-GS, Clinical Dementia Rating – Global Score; CDR-SB, Clinical Dementia Rating – Sum of Boxes; FAQ, Functional Activities Questionnaire; MCI, mild cognitive impairment; MMSE, Mini-Mental State Examination; MOCA, Montreal Cognitive Assessment; NACC, National Alzheimer's Coordinating Center; NACCUDSD, National Alzheimer’s Coordinating Center cognitive status at Uniform Data Set visit; NPI-Q, Neuropsychiatric Inventory – Questionnaire; UDS, Uniform Data Set.

**Supplementary Table 4** Predicted hazard ratios for different age groups, based on model 5

| Base case analysis (classifying disease severity by Green et al. 2016) model 5^a^ | | | |
| --- | --- | --- | --- |
| Disease severity | **Age at start of stage** | **Hazard ratio (95% CI)** | ***P* value** |
| MCI due to AD | 65 | 0.81 (0.42–1.58) | 0.543 |
| Mild AD dementia | 65 | 6.71 (2.66–16.89) | <0.001 |
| Moderate AD dementia | 65 | 14.76 (8.8–24.77) | <0.001 |
| Severe AD dementia | 65 | 30.76 (16.68–56.73) | <0.001 |
| MCI due to AD | 70 | 0.78 (0.47–1.31) | 0.353 |
| Mild AD dementia | 70 | 4.74 (2.37–9.48) | <0.001 |
| Moderate AD dementia | 70 | 8.77 (5.95–12.94) | <0.001 |
| Severe AD dementia | 70 | 18.43 (11.63–29.18) | <0.001 |
| MCI due to AD | 75 | 0.76 (0.52–1.1) | 0.145 |
| Mild AD dementia | 75 | 3.35 (2.06–5.44) | <0.001 |
| Moderate AD dementia | 75 | 5.21 (3.92–6.93) | <0.001 |
| Severe AD dementia | 75 | 11.04 (7.81–15.6) | <0.001 |
| MCI due to AD | 80 | 0.73 (0.55–0.96) | 0.024 |
| Mild AD dementia | 80 | 2.37 (1.68–3.33) | <0.001 |
| Moderate AD dementia | 80 | 3.1 (2.44–3.94) | <0.001 |
| Severe AD dementia | 80 | 6.61 (4.82–9.07) | <0.001 |
|  |  |  |  |
| Sensitivity analysis (classifying disease severity by CDR-SB) model 5^a^ | | | |
| Disease severity | **Age at start of stage** | **Hazard ratio (95% CI)** | ***P* value** |
| MCI due to AD | 65 | 0.74 (0.25–2.15) | 0.576 |
| Mild AD dementia | 65 | 10.58 (4.16–26.93) | <0.001 |
| Moderate AD dementia | 65 | 17.65 (6.58–47.32) | <0.001 |
| Severe AD dementia | 65 | 41.85 (13.32–131.48) | <0.001 |
| MCI due to AD | 70 | 0.7 (0.3–1.63) | 0.415 |
| Disease severity | **Age at start of stage** | **Hazard ratio (95% CI)** | ***P* value** |
| Mild AD dementia | 70 | 6.92 (3.51–13.63) | <0.001 |
| Moderate AD dementia | 70 | 11.87 (5.47–25.76) | <0.001 |
| Severe AD dementia | 70 | 27.81 (11.51–67.23) | <0.001 |
| MCI due to AD | 75 | 0.67 (0.36–1.26) | 0.218 |
| Mild AD dementia | 75 | 4.52 (2.86–7.14) | <0.001 |
| Moderate AD dementia | 75 | 7.98 (4.44–14.35) | <0.001 |
| Severe AD dementia | 75 | 18.48 (9.46–36.13) | <0.001 |
| MCI due to AD | 80 | 0.65 (0.41–1.01) | 0.055 |
| Mild AD dementia | 80 | 2.96 (2.09–4.17) | <0.001 |
| Moderate AD dementia | 80 | 5.37 (3.42–8.43) | <0.001 |
| Severe AD dementia | 80 | 12.29 (6.97–21.66) | <0.001 |

^a^ Model 5 included covariates: disease severity, age, age x severity interaction, sex, BMI, years of education, type of residence, and APOE status.

AD, Alzheimer's Disease; APOE, apolipoprotein E; CDR-SB, Clinical Dementia Rating – Sum of Boxes; CI, confidence interval; MCI, mild cognitive impairment.

**Supplementary Table 5** Sensitivity analysis on relative risks of death estimated by Cox proportional-hazards models

|  | Model 3 without comorbidities | Model 3 with data cut March 1, 2020  (pre Covid-19 lockdowns in US) |
| --- | --- | --- |
| Model parameters, hazard ratios (95% CI) |  |  |
| Disease severity |  |  |
| CN | ref | ref |
| MCI due to AD | 1.03 (0.80–1.33) | 0.95 (0.73–1.24) |
| Mild AD dementia | 2.28*** (1.60–3.26) | 2.34*** (1.64–3.34) |
| Moderate AD dementia | 2.62*** (1.91–3.59) | 2.78*** (2.05–3.77) |
| Severe AD dementia | 7.64*** (5.17–11.3) | 7.74*** (5.26–11.4) |
| Age at start of stage | 1.11*** (1.10–1.13) | 1.11*** (1.09–1.12) |
| Sex |  |  |
| Male | ref | ref |
| Female | 0.59*** (0.50–0.70) | 0.6*** (0.51–0.72) |
| BMI |  |  |
| Normal or healthy weight | ref | ref |
| Underweight | 1.81 (0.98–3.35) | 1.86* (1.02–3.37) |
| Overweight | 0.77** (0.64–0.92) | 0.74** (0.62–0.89) |
| Obese | 1.23 (1.00–1.53) | 1.16 (0.93–1.44) |
| Race |  |  |
| White | ref | ref |
| Black or African American | 0.94 (0.72–1.23) | 0.92 (0.70–1.21) |
| Asia | 0.23* (0.06–0.89) | 0.25* (0.07–0.93) |
| Other | 1.81 (0.58–5.58) | 1.85 (0.60–5.68) |
| Hispanic/Latino ethnicity |  |  |
| No | ref | ref |
| Yes | 0.35** (0.18–0.66) | 0.37** (0.20–0.69) |
| Unknown | 0.58 (0.18–1.91) | 0.5 (0.15–1.74) |
| Years of education | 0.98 (0.95, 1.01) | 0.98 (0.95, 1.01) |
| More than 12 years of education | - | - |
| Less or 12 years | - | - |
| More than 12 years | - | - |
| Type of residence |  |  |
| Single- or multi-family private residence (apartment, condo, house) | ref | ref |
| Retirement community or independent group living | 1.34** (1.10–1.63) | 1.31* (1.07–1.62) |
| Institutionalized | 2.47*** (1.70–3.60) | 2.45*** (1.68–3.55) |
| APOE status |  |  |
| No e4 allele | ref | ref |
| One or two copies of e4 allele | 1.09 (0.91–1.29) | 1.11 (0.93–1.32) |
| Presence or history of CAD |  |  |
| No | - | ref |
| Yes | - | 1.94*** (1.50–2.51) |
| Presence or history of CVD |  |  |
| No | - | ref |
| Yes | - | 1.34* (1.01–1.79) |
| Currently smoking |  |  |
| No | ref | ref |
| Yes | 2.11*** (1.42–3.13) | 2.01** (1.33–3.05) |
| Alcohol abuse |  |  |
| Absent | ref | ref |
| Recent/active | 1.49 (0.69–3.19) | 1.43 (0.64–3.18) |
| Remote/inactive | 1.66** (1.15–2.39) | 1.47 (0.97–2.23) |
| Disease severity by age at start of stage |  |  |
| MCI due to AD by age at index visit | - | - |
| Mild AD by age at index visit | - | - |
| Moderate AD by age at index visit | - | - |
| Severe AD by age at index visit | - | - |
| Number of observations per stage |  |  |
| CN | 8870 | 8900 |
| MCI due to AD | 497 | 501 |
| Mild AD dementia | 367 | 368 |
| Moderate AD dementia | 276 | 276 |
| Severe AD dementia | 53 | 53 |

**p* < 0.05; ***p* < 0.01, ****p* < 0.001.

AD, Alzheimer's disease; APOE, apolipoprotein E; BMI, body mass index; CAD, coronary artery disease; CI, confidence interval; CN, cognitively normal; CVD, cerebrovascular disease; MCI, mild cognitive impairment; ref, reference category.

**Supplementary Table 6** Participant characteristics comparing MCI due to AD cohort with subset with positive AD biomarker

|  | MCI due to AD | Biomarker-positive MCI due to AD |
| --- | --- | --- |
|  | **(*n* = 937)** | **(*n* = 47)** |
| Age at first visit (including pre-index visits) |  |  |
| Mean (SD) | 75.6 (7.78) | 73.1 (7.58) |
| Median [Min, Max] | 76.0 [41.0,102] | 73.0 [55.0, 87.0] |
| Age at study entry (index visit) |  |  |
| Mean (SD) | 79.2 (8.05) | 76.3 (7.71) |
| Median [Min, Max] | 80.0 [51.0, 103] | 76.0 [58.0, 89.0] |
| Sex, *n* (%) |  |  |
| Male | 390 (41.6) | 22 (46.8) |
| Female | 547 (58.4) | 25 (53.2) |
| BMI, *n* (%) |  |  |
| Normal or healthy weight | 358 (38.2) | 18 (38.3) |
| Underweight | 20 (2.1) | 3 (6.4) |
| Overweight | 329 (35.1) | 17 (36.2) |
| Obese | 148 (15.8) | 7 (14.9) |
| Missing | 82 (8.8) | 2 (4.3) |
| Race, *n* (%) |  |  |
| White | 790 (84.3) | 45 (95.7) |
| Black or African American | 114 (12.2) | 2 (4.3) |
| American Indian or Alaska Native | 2 (0.2) | 0 (0) |
| Native Hawaiian or Other Pacific Islander | 0 (0) | 0 (0) |
| Asia | 21 (2.2) | 0 (0) |
| Other | 5 (0.5) | 0 (0) |
| Unknown | 5 (0.5) | 0 (0) |
| Hispanic/Latino ethnicity, *n* (%) |  |  |
| No | 871 (93.0) | 45 (95.7) |
| Yes | 64 (6.8) | 1 (2.1) |
| Unknown | 2 (0.2) | 1 (2.1) |
| Years of education |  |  |
| Mean (SD) | 15.7 (3.33) | 17.2 (2.32) |
| Median [Min, Max] | 16.0 [0, 28.0] | 17.0 [12.0, 23.0] |
| Missing | 5 (0.5) | 0 (0) |
| More than 12 years of education, *n* (%) |  |  |
| Less or 12 years | 179 (19.1) | 3 (6.4) |
| More than 12 years | 753 (80.4) | 44 (93.6) |
| Missing | 5 (0.5) | 0 (0) |
| Type of residence, *n* (%) |  |  |
| Single- or multi-family private residence (apartment, condo, house) | 813 (86.8) | 42 (89.4) |
| Retirement community or independent group living | 85 (9.1) | 4 (8.5) |
| Assisted living, adult family home, or boarding home | 11 (1.2) | 0 (0) |
| Skilled nursing facility, nursing home, hospital, or hospice | 1 (0.1) | 0 (0) |
| Other or unknown | 27 (2.9) | 1 (2.1) |
| Currently smoking, *n* (%) |  |  |
| No | 580 (61.9) | 17 (36.2) |
| Yes | 15 (1.6) | 0 (0) |
| Missing | 342 (36.5) | 30 (63.8) |
| Alcohol abuse, *n* (%) |  |  |
| Absent | 572 (61.0) | 17 (36.2) |
| Recent/active | 3 (0.3) | 0 (0) |
| Remote/inactive | 16 (1.7) | 0 (0) |
| Missing | 346 (36.9) | 30 (63.8) |
| APOE status, *n* (%) |  |  |
| No e4 allele | 530 (56.6) | 24 (51.1) |
| One or two copies of e4 allele | 323 (34.5) | 20 (42.6) |
| Missing | 84 (9.0) | 3 (6.4) |
| Presence or history of CAD, *n* (%) |  |  |
| No | 856 (91.4) | 44 (93.6) |
| Yes | 81 (8.6) | 3 (6.4) |
| Missing | - | - |
| Presence or history of CVD, *n* (%) |  |  |
| No | 871 (93.0) | 45 (95.7) |
| Yes | 66 (7.0) | 2 (4.3) |
| Missing | - | - |
| CDR-GS at index date |  |  |
| Mean (SD) | 0.415 (0.188) | 0.372 (0.220) |
| Median [Min, Max] | 0.500 [0, 0.500] | 0.500 [0, 0.500] |
| MMSE/MOCA at index date |  |  |
| Mean (SD) | 27.7 (2.16) | 28.0 (2.16) |
| Median [Min, Max] | 28.0 [18.0, 30.0] | 29.0 [22.0, 30.0] |
| Missing | 56 (6.0) | 2 (4.3) |
| FAQ score at index date |  |  |
| Mean (SD) | 1.95 (3.22) | 1.80 (3.36) |
| Median [Min, Max] | 0 [0, 19.0] | 0 [0, 12.0] |
| Missing | 31 (3.3) | 2 (4.3) |
| NPI-Q at index date |  |  |
| Mean (SD) | 1.54 (2.63) | 1.43 (2.99) |
| Median [Min, Max] | 0 [0, 20.0] | 0 [0, 16.0] |
| AD biomarker positive |  |  |
| No | - | - |
| Yes | - | - |
| Missing | - | - |
| Number of visits (in this stage) |  |  |
| Mean (SD) | - | - |
| Median [Min, Max] | - | - |
| Number of visits (overall) |  |  |
| Mean (SD) | 7.29 (3.44) | 7.26 (3.44) |
| Median [Min, Max] | 7.00 [2.00, 16.0] | 7.00 [2.00, 14.0] |

AD, Alzheimer's disease; APOE, apolipoprotein E; BMI, body mass index; CAD, coronary artery disease; CDR-GS, Clinical Dementia Rating – Global Score; CVD, cerebrovascular disease; FAQ, Functional Activities Questionnaire; MCI, mild cognitive impairment; MMSE, Mini-Mental State Examination; MOCA, Montreal Cognitive Assessment; NPI-Q, Neuropsychiatric Inventory – Questionnaire; SD, standard deviation.

**Supplementary Table 7** Sensitivity analysis of relative risks of death estimated by Cox proportional-hazards models (classifying disease severity by CDR-SB)

|  | Model 1  Crude | Model 2  Parsimonious | Model 3  Exploratory | Model 4  Lasso + sex | Model 5  Age x severity interaction |  |
| --- | --- | --- | --- | --- | --- | --- |
| Model parameters, hazard ratios (95% CI) |  |  |  |  |  |  |
| Disease severity |  |  |  |  |  |  |
| CN | ref | ref | ref | ref | ref |  |
| MCI due to AD | 1.68** (1.21–2.34) | 0.63** (0.46–0.88) | 0.8 (0.53–1.21) | 0.66* (0.46–0.95) | 1.3 (0.02–93.3) |  |
| Mild AD dementia | 6.1*** (4.67–7.99) | 2.43*** (1.81–3.26) | 2.59*** (1.50–4.46) | 2.54*** (1.76–3.67) | 2659*** (27.5–257,493) |  |
| Moderate AD dementia | 11.5*** (8.27–15.9) | 3.77*** (2.66–5.34) | 3.51*** (1.96–6.29) | 4.05*** (2.56–6.42) | 3070*** (53.8–175,038) |  |
| Severe AD dementia | 25.6*** (17.5–37.4) | 8.53*** (5.45–13.3) | 9.3*** (3.72–23.3) | 10.7*** (5.51–20.8) | 8477*** (48.9–1,471,122) |  |
| Age at start of stage | - | 1.12*** (1.11–1.13) | 1.13*** (1.11–1.14) | 1.12*** (1.11–1.13) | 1.13*** (1.12–1.15) |  |
| Sex |  |  |  |  |  |  |
| Male | - | ref | ref | ref | ref |  |
| Female | - | 0.66*** (0.57–0.76) | 0.64*** (0.53–0.77) | 0.62*** (0.52–0.74) | 0.63*** (0.53–0.75) |  |
| BMI |  |  |  |  |  |  |
| Normal or healthy weight | - | - | ref | ref | ref |  |
| Underweight | - | - | 1.33 (0.55–3.22) | 1.51 (0.64–3.55) | 1.37 (0.57–3.32) |  |
| Overweight | - | - | 0.78* (0.63–0.96) | 0.79* (0.65–0.96) | 0.77* (0.64–0.94) |  |
| Obese | - | - | 1.37** (1.09–1.72) | 1.4** (1.13–1.73) | 1.4** (1.14–1.74) |  |
| Race |  |  |  |  |  |  |
| White | - | - | ref | - | - |  |
| Black or African American | - | - | 0.95 (0.71–1.27) | - | - |  |
| Asia | - | - | 0.18 (0.02–1.26) | - | - |  |
| Other | - | - | 1.87 (0.67–5.23) | - | - |  |
| Hispanic/Latino ethnicity |  |  |  |  |  |  |
| No | - | - | ref | - | - |  |
| Yes | - | - | 0.45* (0.24–0.84) | - | - |  |
| Unknown | - | - | 0.79 (0.07–9.56) | - | - |  |
| Years of education | **-** | - | 0.98 (0.94–1.01) | - | - |  |
| More than 12 years of education | **-** | - | **-** | **-** | **-** |  |
| Less or 12 years | - | ref | - | ref | ref |  |
| More than 12 years | - | 0.83 (0.69–1.01) | - | 0.86 (0.69–1.08) | 0.89 (0.71–1.12) |  |
| Type of residence |  |  |  |  |  |  |
| Single- or multi-family private residence (apartment, condo, house) | - | - | ref | ref | ref |  |
| Retirement community or independent group living | - | - | 1.3* (1.03–1.65) | 1.38** (1.10–1.72) | 1.32* (1.06–1.65) |  |
| Institutionalized | - |  | 2.82*** (1.73–4.59) | 2.97*** (1.95–4.54) | 3.06*** (2.04–4.58) |  |
| APOE status |  | - |  |  |  |  |
| No e4 allele | - | - | ref | ref | ref |  |
| 1 or 2 copies of e4 allele | - | - | 1.12 (0.92–1.36) | 1.09 (0.90–1.32) | 1.02 (0.84–1.24) |  |
| Presence or history of CAD |  |  |  |  |  |  |
| No | - | - | ref | - | - |  |
| Yes | - | - | 1.74*** (1.28–2.37) | - | - |  |
| Presence or history of CVD |  |  |  |  |  |  |
| No | - | - | ref | - | - |  |
| Yes | - | - | 1.21 (0.86-1.70) | - | - |  |
| Currently smoking |  |  |  |  | - |  |
| No | - | - | ref | - | - |  |
| Yes | - | - | 2.33*** (1.51–3.58) | - | - |  |
| Alcohol abuse |  |  |  |  |  |  |
| Absent | - | - | ref | - | - |  |
| Recent/active | - | - | 1.54 (0.52–4.58) | - | - |  |
| Remote/inactive | - | - | 1.4 (0.84–2.33) | - | - |  |
| Disease severity by age at start of stage |  |  |  |  |  |  |
| MCI due to AD by age at index visit | - | - | - | - | 0.99 (0.94–1.04) |  |
| Mild AD by age at index visit | - | - | - | - | 0.92** (0.87–0.97) |  |
| Moderate AD by age at index visit | - | - | - | - | 0.92** (0.88–0.97) |  |
| Severe AD by age at index visit | - | - | - | - | 0.92* (0.86–0.98) |  |
| Number of observations per stage |  |  |  |  |  |  |
| CN | 11,458 | 11,395 | 8859 | 8971 | 8971 |  |
| MCI due to AD | 415 | 411 | 214 | 342 | 342 |  |
| Mild AD dementia | 405 | 404 | 115 | 247 | 247 |  |
| Moderate AD dementia | 125 | 125 | 39 | 69 | 69 |  |
| Severe AD dementia | 45 | 45 | 4 | 14 | 14 |  |

**p* < 0.05; ***p* < 0.01, ****p* < 0.001

AD, Alzheimer's disease; APOE, apolipoprotein E; BMI, body mass index; CAD, coronary artery disease; CI, confidence interval; CN, cognitively normal; CVD, cerebrovascular disease; MCI, mild cognitive impairment; ref, reference category.

**Supplementary Table 8** Participant characteristics of complete cases contributing to each model (including multiple observations from some participants included in several cohorts if progressing)

|  | Model 1: crude  *n* = 13,459 | Model 2: parsimony (age + sex + education)  *n* = 13,389 | Model 3: exploratory (all variables)  *n* = 10,052 | Model 4:  lasso + sex  *n* = 10,668 | Model 5:  Age x severity interaction  *n* = 10,668 |  |
| --- | --- | --- | --- | --- | --- | --- |
| Age at first visit (including pre-index visits) |  |  |  |  |  |  |
| Mean (SD) | 71.2 (9.14) | 71.2 (9.12) | 71.0 (8.97) | 71.1 (9.01) | 71.1 (9.01) |  |
| Median [Min, Max] | 71.0 [38.0, 104] | 71.0 [38.0, 104] | 71.0 [41.0, 102] | 71.0 [38.0, 102] | 71.0 [38.0, 102] |  |
| Age at study entry (index visit) |  |  |  |  |  |  |
| Mean (SD) | 71.7 (9.42) | 71.7 (9.41) | 71.4 (9.17) | 71.7 (9.32) | 71.7 (9.32) |  |
| Median [Min, Max] | 72.0 [50.0, 104] | 72.0 [50.0, 104] | 71.0 [50.0, 102] | 72.0 [50.0, 103] | 72.0 [50.0, 103] |  |
| Sex, *n* (%) |  |  |  |  |  |  |
| Male | 4775 (35.5) | 4752 (35.5) | 3601 (35.8) | 3862 (36.2) | 3862 (36.2) |  |
| Female | 8684 (64.5) | 8637 (64.5) | 6806 (63.8) | 6806 (63.8) | 6851 (63.8) |  |
| BMI, *n* (%) |  |  |  |  |  |  |
| Normal or healthy weight | 4488 (33.3) | 4465 (33.3) | 3552 (35.3) | 3807 (35.7) | 3807 (35.7) |  |
| Underweight | 168 (1.2) | 167 (1.2) | 123 (1.2) | 139 (1.3) | 139 (1.3) |  |
| Overweight | 4787 (35.6) | 4769 (35.6) | 3853 (38.3) | 4069 (38.1) | 4069 (38.1) |  |
| Obese | 3221 (23.9) | 3209 (24.0) | 2524 (25.1) | 2653 (24.9) | 2653 (24.9) |  |
| Missing | 795 (5.9) | 779 (5.8) | 0 (0) | 0 (0) | 0 (0) |  |
| Race |  |  |  |  |  |  |
| White | 10,968 (81.5) | 10,911 (81.5) | 8431 (83.9) | 8916 (83.6) | 8916 (83.6) |  |
| Black or African American | 1904 (14.1) | 1899 (14.2) | 1286 (12.8) | 1361 (12.8) | 1361 (12.8) |  |
| Asia | 324 (2.4) | 319 (2.4) | 219 (2.2) | 236 (2.2) | 236 (2.2) |  |
| Other | 191 (1.4) | 190 (1.4) | 116 (1.2) | 118 (1.1) | 118 (1.1) |  |
| Unknown | 72 (0.5) | 70 (0.5) | 0 (0) | 37 (0.3) | 37 (0.3) |  |
| Hispanic/Latino ethnicity, *n* (%) |  |  |  |  |  |  |
| No | 12,506 (92.9) | 12,445 (92.9) | 9444 (94.0) | 10,007 (93.8) | 10,007 (93.8) |  |
| Yes | 891 (6.6) | 887 (6.6) | 575 (5.7) | 628 (5.9) | 628 (5.9) |  |
| Unknown | 62 (0.5) | 57 (0.4) | 33 (0.3) | 33 (0.3) | 33 (0.3) |  |
| Years of education, *n* (%) |  |  |  |  |  |  |
| Mean (SD) | 15.8 (2.99) | 15.8 (2.99) | 15.9 (2.92) | 15.9 (2.93) | 15.9 (2.93) |  |
| Median [Min, Max] | 16.0 [0, 30.0] | 16.0 [0, 30.0] | 16.0 [0, 30.0] | 16.0 [0, 30.0] | 16.0 [0, 30.0] |  |
| Missing | 70 (0.5) | 0 (0) | 0 (0) | 0 (0) | 0 (0) |  |
| More than 12 years of education, *n* (%) |  |  |  |  |  |  |
| Less or 12 years | 2259 (16.8) | 2259 (16.9) | 1616 (16.1) | 1723 (16.2) | 1723 (16.2) |  |
| More than 12 years | 11,130 (82.7) | 11,130 (83.1) | 8436 (83.9) | 8945 (83.8) | 8945 (83.8) |  |
| Missing | 70 (0.5) | 0 (0) | 0 (0) | 0 (0) | 0 (0) |  |
| Type of residence, *n* (%) |  |  |  |  |  |  |
| Single- or multi-family private residence (apartment, condo, house) | 12,443 (92.5) | 12,380 (92.5) | 9374 (93.3) | 9939 (93.2) | 9939 (93.2) |  |
| Retirement community or independent group living | 710 (5.3) | 709 (5.3) | 529 (5.2) | 571 (5.3) | 571 (5.3) |  |
| Institutionalized | 87 (0.6) | 85 (0.6) | 45 (0.4) | 52 (0.5) | 52 (0.5) |  |
| Other or unknown | 219 (1.6) | 215 (1.6) | 104 (1.0) | 106 (1.0) | 106 (1.0) |  |
| Currently smoking, *n* (%) |  |  |  |  |  |  |
| No | 12,293 (91.3) | 12,227 (91.3) | 9695 (96.4) | 9752 (91.4) | 9752 (91.4) |  |
| Yes | 470 (3.5) | 470 (3.5) | 357 (3.6) | 358 (3.4) | 358 (3.4) |  |
| Missing | 696 (5.2) | 692 (5.2) | 0 (0) | 558 (5.2) | 558 (5.2) |  |
| Alcohol abuse, *n* (%) |  |  |  |  |  |  |
| Absent | 12,354 (91.8) | 12,288 (91.8) | 9714 (96.6) | 9781 (91.7) | 9781 (91.7) |  |
| Recent/active | 56 (0.4) | 56 (0.4) | 47 (0.5) | 48 (0.5) | 48 (0.5) |  |
| Remote/inactive | 365 (2.7) | 364 (2.7) | 291 (2.9) | 292 (2.7) | 292 (2.7) |  |
| Missing | 684 (5.1) | 681 (5.1) | 0 (0) | 547 (5.1) | 547 (5.1) |  |
| APOE status, *n* (%) |  |  |  |  |  |  |
| No e4 allele | 7537 (56.0) | 7498 (56.0) | 6672 (66.4) | 6990 (65.5) | 6990 (65.5) |  |
| One or two copies of e4 allele | 3892 (28.9) | 3871 (28.9) | 3380 (33.6) | 3678 (34.5) | 3678 (34.5) |  |
| Missing | 2030 (15.1) | 2020 (15.1) | 0 (0) | 0 (0) | 0 (0) |  |
| Presence or history of CAD, *n* (%) |  |  |  |  |  |  |
| No | 12,754 (94.8) | 12,684 (94.7) | 9590 (95.4) | 10,132 (95.0) | 10,132 (95.0) |  |
| Yes | 683 (5.1) | 683 (5.1) | 462 (4.6) | 523 (4.9) | 523 (4.9) |  |
| Missing | 22 (0.2) | 22 (0.2) | 0 (0) | 13 (0.1) | 13 (0.1) |  |
| Presence or history of CVD, *n* (%) |  |  |  |  |  |  |
| No | 12,762 (94.8) | 12,694 (94.8) | 9537 (94.9) | 10,111 (94.8) | 10,111 (94.8) |  |
| Yes | 695 (5.2) | 693 (5.2) | 515 (5.1) | 557 (5.2) | 557 (5.2) |  |
| Missing | 2 (0.0) | 2 (0.0) | 0 (0) | 0 (0) | 0 (0) |  |
| CDR-GS at index date, *n* (%) |  |  |  |  |  |  |
| Mean (SD) | 0.0968 (0.284) | 0.0970 (0.284) | 0.0785 (0.255) | 0.103 (0.288) | 0.103 (0.288) |  |
| Median [Min, Max] | 0 [0, 3.00] | 0 [0, 3.00] | 0 [0, 3.00] | 0 [0, 3.00] | 0 [0, 3.00] |  |
| MMSE/MOCA at index date, *n* (%) |  |  |  |  |  |  |
| Mean (SD) | 28.4 (2.81) | 28.4 (2.81) | 28.5 (2.55) | 28.3 (2.84) | 28.3 (2.84) |  |
| Median [Min, Max] | 29.0 [2.00, 30.0] | 29.0 [2.00, 30.0] | 29.0 [3.00, 30.0] | 29.0 [3.00, 30.0] | 29.0 [3.00, 30.0] |  |
| Missing | 278 (2.1) | 247 (1.8) | 156 (1.6) | 160 (1.5) | 160 (1.5) |  |
| FAQ score at index date, *n* (%) |  |  |  |  |  |  |
| Mean (SD) | 1.44 (4.67) | 1.44 (4.66) | 1.22 (4.29) | 1.49 (4.73) | 1.49 (4.73) |  |
| Median [Min, Max] | 0 [0, 30.0] | 0 [0, 30.0] | 0 [0, 30.0] | 0 [0, 30.0] | 0 [0, 30.0] |  |
| Missing | 381 (2.8) | 376 (2.8) | 221 (2.2) | 236 (2.2) | 236 (2.2) |  |
| NPI-Q at index date, *n* (%) |  |  |  |  |  |  |
| Mean (SD) | 0.972 (2.21) | 0.974 (2.22) | 0.903 (2.07) | 0.977 (2.21) | 0.977 (2.21) |  |
| Median [Min, Max] | 0 [0, 28.0] | 0 [0, 28.0] | 0 [0, 27.0] | 0 [0, 28.0] | 0 [0, 28.0] |  |
| AD biomarker positive, *n* (%) | |  |  |  |  |  |
| No | 782 (5.8) | 779 (5.8) | 631 (6.3) | 664 (6.2) | 664 (6.2) |  |
| Yes | 350 (2.6) | 349 (2.6) | 221 (2.2) | 302 (2.8) | 302 (2.8) |  |
| Missing | 12,327 (91.6) | 12,261 (91.6) | 9200 (91.5) | 9702 (90.9) | 9702 (90.9) |  |
| Number of visits (in this stage), *n* (%) | |  |  |  |  |  |
| Mean (SD) | 3.64 (3.06) | 3.65 (3.06) | 4.08 (3.19) | 3.97 (3.14) | 3.97 (3.14) |  |
| Median [Min, Max] | 3.00 [1.00, 16.0] | 3.00 [1.00, 16.0] | 3.00 [1.00, 16.0] | 3.00 [1.00, 16.0] | 3.00 [1.00, 16.0] |  |
| Number of visits (overall), *n* (%) |  |  |  |  |  |  |
| Mean (SD) | 5.18 (3.55) | 5.18 (3.55) | 5.60 (3.52) | 5.65 (3.53) | 5.65 (3.53) |  |
| Median [Min, Max] | 4.00 [1.00, 16.0] | 4.00 [1.00, 16.0] | 5.00 [1.00, 16.0] | 5.00 [1.00, 16.0] | 5.00 [1.00, 16.0] |  |

AD, Alzheimer's disease; APOE, apolipoprotein E; BMI, body mass index; CAD, coronary artery disease; CDR-GS, Clinical Dementia Rating – Global Score; CVD, cerebrovascular disease; FAQ, Functional Activities Questionnaire MMSE, Mini-Mental State Examination; MOCA, Montreal Cognitive Assessment; NPI-Q, Neuropsychiatric Inventory – Questionnaire; SD, standard deviation**.**

**Supplementary Table 9** Participant Characteristics Comparing Those Lost to Follow-Up and Those That Died, Progressed or Continued Until Data Cut (Including Multiple Observations from Some Participants Included in Several Cohorts if Progressing)

|  | Lost to follow-up | |
| --- | --- | --- |
|  | No  (*n* = 6766) | Yes  (*n* = 6693) |
| Age at first visit (including pre-index visits) |  |  |
| Mean (SD) | 72.8 (9.06) | 69.5 (8.92) |
| Median (Q1, Q3) | 73.0 (67.0, 79.0) | 70.0 (64.0, 76.0) |
| Min, Max | 41.0, 104 | 38.0, 100 |
| Age at study entry (index visit) |  |  |
| Mean (SD) | 73.4 (9.37) | 70.0 (9.16) |
| Median (Q1, Q3) | 73.0 (67.0, 80.0) | 70.0 (64.0, 76.0) |
| Min, Max | 50.0, 104 | 50.0, 100 |
| Sex, *n* (%) |  |  |
| Male | 2607 (38.5) | 2168 (32.4) |
| Female | 4159 (61.5) | 4525 (67.6) |
| BMI, *n* (%) |  |  |
| Normal or healthy weight | 2348 (34.7) | 2140 (32.0) |
| Underweight | 87 (1.3) | 81 (1.2) |
| Overweight | 2395 (35.4) | 2392 (35.7) |
| Obese | 1532 (22.6) | 1689 (25.2) |
| Missing | 404 (6.0) | 391 (5.8) |
| Race, *n* (%) |  |  |
| White | 5667 (83.8) | 5301 (79.2) |
| Black or African American | 860 (12.7) | 1044 (15.6) |
| American Indian or Alaska Native | 37 (0.5) | 43 (0.6) |
| Native Hawaiian or Other Pacific Islander | 5 (0.1) | 6 (0.1) |
| Asia | 136 (2.0) | 188 (2.8) |
| Other | 33 (0.5) | 67 (1.0) |
| Unknown | 28 (0.4) | 44 (0.7) |
| Hispanic/Latino ethnicity, *n* (%) |  |  |
| No | 6340 (93.7) | 6166 (92.1) |
| Yes | 402 (5.9) | 489 (7.3) |
| Unknown | 24 (0.4) | 38 (0.6) |
| Years of education, *n* (%) |  |  |
| Mean (SD) | 15.9 (2.91) | 15.8 (3.06) |
| Median (Q1, Q3) | 16.0 (14.0, 18.0) | 16.0 (14.0, 18.0) |
| Min, Max | 0, 29.0 | 0, 30.0 |
| Missing | 29 (0.4) | 41 (0.6) |
| More than 12 years of education, *n* (%) |  |  |
| Less or 12 years | 1066 (15.8) | 1193 (17.8) |
| More than 12 years | 5671 (83.8) | 5459 (81.6) |
| Missing | 29 (0.4) | 41 (0.6) |
| Type of residence, *n* (%) |  |  |
| Single- or multi-family private residence (apartment, condo, house) | 6154 (91.0) | 6289 (94.0) |
| Retirement community or independent group living | 426 (6.3) | 284 (4.2) |
| Institutionalized | 66 (1.0) | 21 (0.3) |
| Other or unknown | 120 (1.8) | 99 (1.5) |
| Currently smoking, *n* (%) |  |  |
| No | 6169 (91.2) | 6124 (91.5) |
| Yes | 209 (3.1) | 261 (3.9) |
| Missing | 388 (5.7) | 308 (4.6) |
| Alcohol abuse, *n* (%) |  |  |
| Absent | 6158 (91.0) | 6196 (92.6) |
| Recent/active | 31 (0.5) | 25 (0.4) |
| Remote/inactive | 195 (2.9) | 170 (2.5) |
| Missing | 382 (5.6) | 302 (4.5) |
| APOE status, *n* (%) |  |  |
| No e4 allele | 3866 (57.1) | 3671 (54.8) |
| One or two copies of e4 allele | 2039 (30.1) | 1853 (27.7) |
| Missing | 861 (12.7) | 1169 (17.5) |
| Presence or history of CAD, *n* (%) |  |  |
| No | 6366 (94.1) | 6388 (95.4) |
| Yes | 392 (5.8) | 291 (4.3) |
| Missing | 8 (0.1) | 14 (0.2) |
| Presence or history of CVD, *n* (%) |  |  |
| No | 6358 (94.0) | 6404 (95.7) |
| Yes | 408 (6.0) | 287 (4.3) |
| Missing | 0 (0) | 2 (0.0) |
| CDR-GS at index date |  |  |
| Mean (SD) | 0.115 (0.318) | 0.0784 (0.244) |
| Median (Q1, Q3) | 0 (0, 0) | 0 (0, 0) |
| Min, Max | 0, 3.00 | 0, 2.00 |
| MMSE/MOCA at index date |  |  |
| Mean (SD) | 28.2 (2.99) | 28.5 (2.60) |
| Median (Q1, Q3) | 29.0 (28.0, 30.0) | 29.0 (28.0, 30.0) |
| Min, Max | 2.00, 30.0 | 6.00, 30.0 |
| Missing | 126 (1.9) | 152 (2.3) |
| FAQ score at index date |  |  |
| Mean (SD) | 1.76 (5.21) | 1.12 (4.01) |
| Median (Q1, Q3) | 0 (0, 0) | 0 (0, 0) |
| Min, Max | 0, 30.0 | 0, 30.0 |
| Missing | 170 (2.5) | 211 (3.2) |
| NPI-Q at index date |  |  |
| Mean (SD) | 1.06 (2.31) | 0.880 (2.10) |
| Median (Q1, Q3) | 0 (0, 1.00) | 0 (0, 1.00) |
| Min, Max | 0, 26.0 | 0, 28.0 |
| AD biomarker positive, *n* (%) |  |  |
| No | 475 (7.0) | 307 (4.6) |
| Yes | 240 (3.5) | 110 (1.6) |
| Missing | 6051 (89.4) | 6276 (93.8) |
| Number of visits (in this stage) |  |  |
| Mean (SD) | 3.78 (3.17) | 3.50 (2.92) |
| Median (Q1, Q3) | 3.00 (1.00, 5.00) | 2.00 (1.00, 5.00) |
| Min, Max | 1.00, 16.0 | 1.00, 15.0 |
| Number of visits (overall) |  |  |
| Mean (SD) | 6.21 (3.63) | 4.14 (3.14) |
| Median (Q1, Q3) | 6.00 (3.00, 9.00) | 3.00 (2.00, 6.00) |
| Min, Max | 1.00, 16.0 | 1.00, 15.0 |

AD, Alzheimer's disease; APOE, apolipoprotein E; BMI, body mass index; CAD, coronary artery disease; CDR-GS, Clinical Dementia Rating – Global Score; CVD, cerebrovascular disease; FAQ, Functional Activities Questionnaire; MMSE, Mini-Mental State Examination; MOCA, Montreal Cognitive Assessment; NPI-Q, Neuropsychiatric Inventory – Questionnaire; Q1, first quartile; Q3, third quartile; SD, standard deviation**.**
